# Highly stereocontrolled synthesis of *trans*-enediynes via carbocupration of fluoroalkylated diynes

**DOI:** 10.3762/bjoc.8.249

**Published:** 2012-12-19

**Authors:** Tsutomu Konno, Misato Kishi, Takashi Ishihara

**Affiliations:** 1 Department of Chemistry and Materials Technology, Kyoto Institute of Technology, Matsugasaki, Sakyo-ku, Kyoto 606-0962, Japan

**Keywords:** carbocupration, carbometallation, diyne, enediyne, fluorine, highly regioselective, highly stereoselective

## Abstract

Treatment of readily prepared (*Z*)-6-benzyloxy-1,1,1,2-tetrafluoro-6-methyl-2-hepten-4-yne with 1.5 equiv of LHMDS in −78 °C for 1 h gave the corresponding trifluoromethylated diyne in an excellent yield. This diyne was found to be a good substrate for the carbocupration with various higher-ordered cyanocuprates to give the corresponding vinylcuprates in a highly regio- and stereoselective manner. The in situ generated vinylcuprates could react very smoothly with an excess amount of iodine, the vinyl iodides being obtained in high yields. Thus-obtained iodides underwent a very smooth Sonogashira cross-coupling reaction to afford various *trans*-enediynes in high yields.

## Introduction

**Figure 1:**
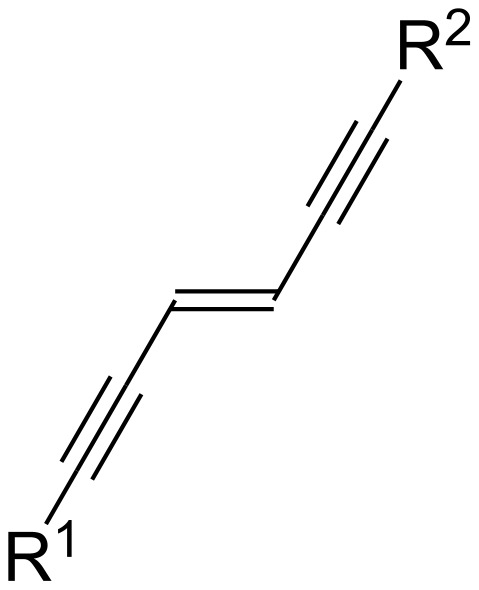
*trans*-Enediyne.

*trans*-Enediynes (*trans*-hex-3-ene-1,5-diynes), as shown in Figure 1, are well-recognized as one of the most important building blocks because they are frequently utilized for the synthesis of π-conjugated polymers, which have attracted much attention in the fields of electronic and photonic materials science [1–3].

While numerous synthetic approaches to non-fluorinated *trans*-enediynes have been reported so far, there has been quite a limited number studies on the preparation of fluoroalkylated *trans*-enediynes [4–7], although the introduction of fluorine atom(s) into organic molecules very often changes their physical as well as chemical characteristics significantly, resulting in the discovery of new materials with unique physical properties [8–14].

**Scheme 1:**
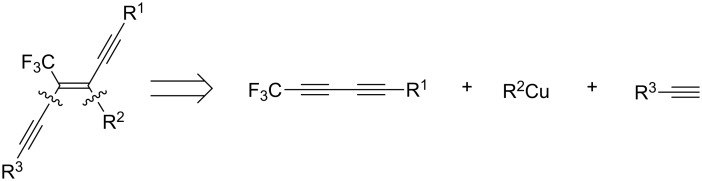
Synthetic strategy for the preparation of trifluoromethylated diynes.

In this paper we report a convenient and efficient access to trifluoromethylated enediynes by the highly regio- and stereoselective carbocupration reaction of trifluoromethylated diyne with various organocuprates (Scheme 1).

## Results and Discussion

**Scheme 2:**
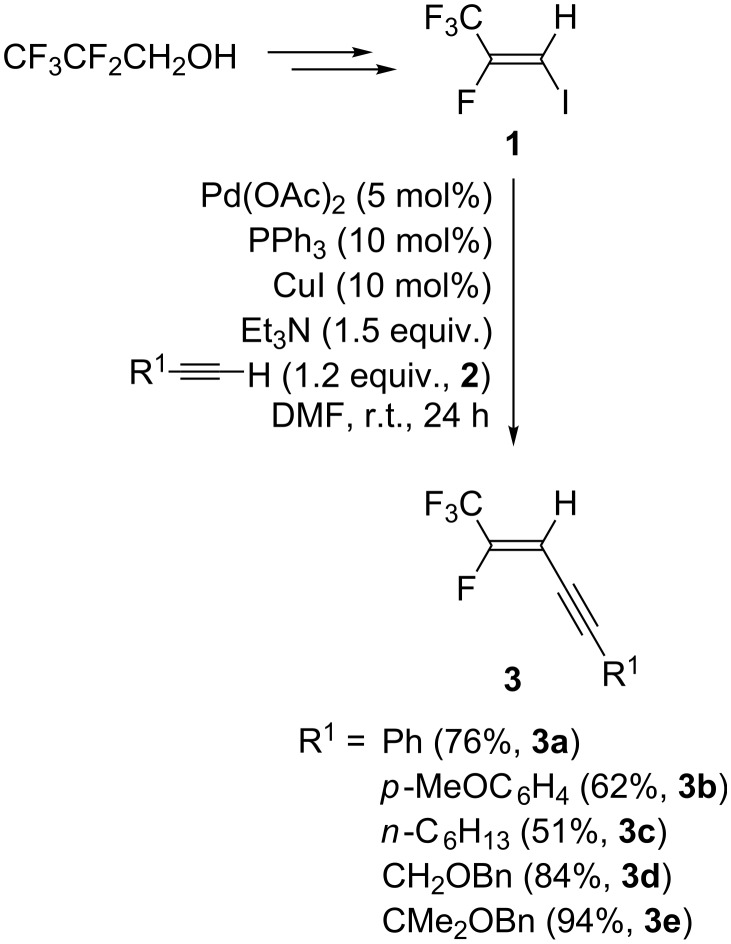
Preparation of various enynes.

Our initial studies began with the preparation of trifluoromethylated diyne derivatives [15–22]. Thus, treatment of 2,3,3,3-tetrafluoro-1-iodo-1-propene (**1**), which could be easily prepared from 2,2,3,3,3-pentafluoropropanol in three steps [23], with 1.2 equiv of terminal alkynes **2** and 1.5 equiv of Et_3_N in the presence of 5 mol % of Pd(OAc)_2_ and 10 mol % each of PPh_3_ and CuI in DMF at room temperature for 24 h, gave the corresponding Sonogashira cross-coupling products **3a**–**e** in good to high yields [24–26] (Scheme 2).

Subsequently, **3a** was subjected to the usual β-elimination conditions according to the literature [23,27–29], i.e., treatment of **3a** with 1.5 equiv of potassium *tert*-butoxide (*t*-BuOK) in THF at room temperature for 2 h. Very surprisingly, the desired trifluoromethylated diyne **4a** was not detected at all and the addition–elimination product **5a** was obtained quantitatively. Therefore, we examined the reaction conditions for the β-elimination of **3a** in detail (Table 1). As shown in Table 1, entries 2 and 3, the reactions with *t*-BuOK in various solvents, such as 1,4-dioxane and ether, were found to be fruitless, leading to a quantitative formation of **5a**. Changing the base from *t*-BuOK to KOH brought about better results (Table 1, entries 4–6). Thus, the reaction with 1.5 equiv of KOH at the reflux temperature of THF produced the desired diyne **4a** in ca. 10%, while no desired product was given at room temperature. On the other hand, a dramatic change could be observed when the amide bases were used. The use of lithium diisopropylamide (LDA) in THF at −78 °C for 2 h resulted in a significant increase of the yield from 10% to 46% (Table 1, entry 7). Switching LDA into lithium hexamethyldisilazide (LHMDS) led to a further improvement of the yield (Table 1, entry 8). Finally, the best yield was obtained when the reaction was carried out in THF at −78 °C for 1 h by using LHMDS. In this case the desired diyne **4a** was generated in 74% ^19^F NMR yield as a sole product. Unfortunately, **4a** was found to be somewhat thermally unstable, and a partial decomposition was observed in silica-gel column chromatography, **4a** being isolated in very low yield. Additionally, such a partial decomposition of **4a** was also observed even when **4a** was kept in a freezer.

**Table 1:** Investigation of the reaction conditions.

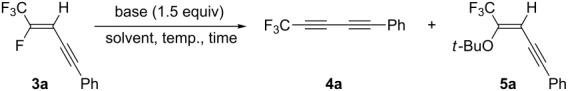							

entry	base	solvent	temp. (°C)	time (h)	yield^a^ (% of **4a)**	yield^a^ (% of **5a)**	recovery^a^ (% of **3a)**

1	*t*-BuOK	THF	rt	2	0	quant.	0
2	*t*-BuOK	1,4-dioxane	rt	2	0	quant.	0
3	*t*-BuOK	Et_2_O	rt	2	0	quant.	0
4	KOH	THF	rt	2	0	—	quant.
5	KOH	THF	reflux	2	9	—	83
6	KOH	THF	reflux	2	10	—	47
7	LDA	THF	−78	2	46	—	0
8	LHMDS	THF	−78	2	69	—	0
9	LHMDS	THF	−78	1	74	—	0

^a^Determined by ^19^F NMR.

With the thus-obtained optimum reaction conditions, we next investigated the β-elimination reaction of various enynes as described in Table 2. As shown in Table 2, entry 2, changing a phenyl group into an anisyl group in R^1^ resulted in a significant increase of the yield from 74% to a quantitative yield. In this case, it was found that **4b** was slightly thermally stable, compared to **4a**, while it could not be isolated in a pure form. In the case of the enyne having an *n*-C_6_H_13_ group as R^1^, on the other hand, the starting material was not completely consumed, and an inseparable mixture of the desired diyne **4c** and the enyne **3c** was given (Table 2, entry 3). In addition, the reaction proceeded very sluggishly in the enyne having CH_2_OBn as R^1^, and neither **3d** nor **4d** could be obtained in high yields (Table 2, entry 4). Interestingly, the enyne **3e** having a CMe_2_OBn group as R^1^ was found to be a good substrate, the desired diyne **4e** being obtained quantitatively (Table 2, entry 5). Additionally, **4e** was so thermally stable that it could be obtained in 95% isolated yield after the silica-gel column chromatography.

**Table 2:** β-Elimination of various enynes.

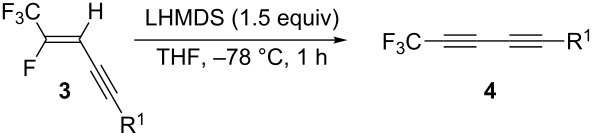			

entry	R^1^	yield^a^ (% of **4**)	recovery^a^ (/% of **3**)

1	Ph (**a**)	74	0
2	*p*-MeOC_6_H_4_ (**b**)	quant.	0
3^b^	*n*-C_6_H_13_ (**c**)	48	35
4	CH_2_OBn (**d**)	3	13
5	CMe_2_OBn (**e**)	quant. (95)	0

^a^Determined by ^19^F NMR. Value in parentheses is of isolated yield. ^b^Carried out for 24 h.

**Figure 2:**
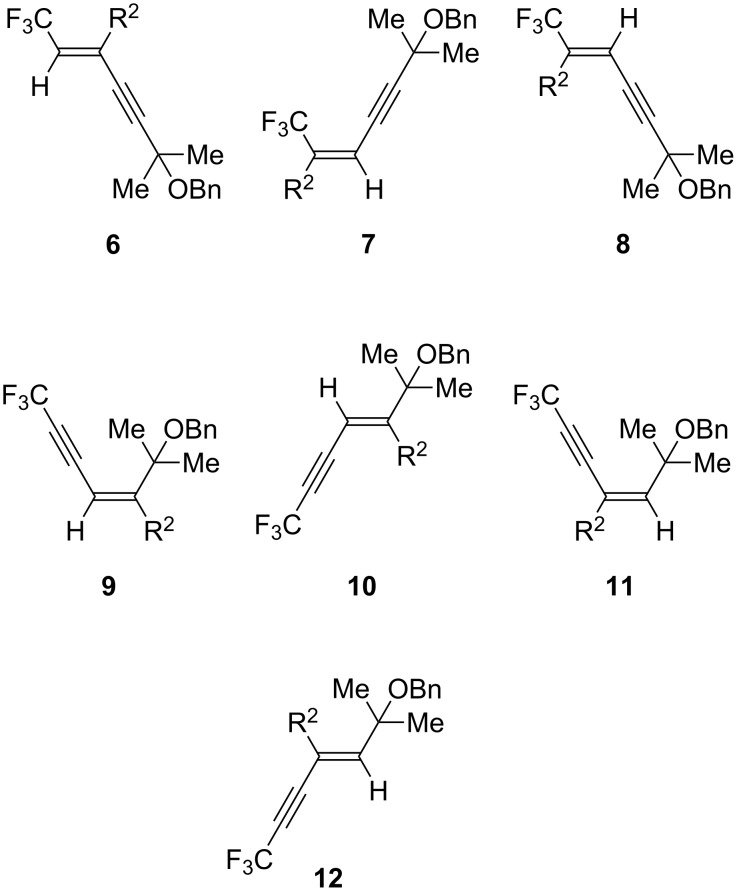
Regio- and stereoisomers.

With the substrate **4e** in hand, our interest was next directed toward the carbocupration reaction of **4e**. First of all, we attempted the investigation of the reaction conditions for the carbocupration reaction of **4e**, as described in Table 3. Thus, treatment of **4e** with 1.2 equiv of higher-ordered cyanocuprate (*n*-Bu)_2_CuLi·LiCN, which was prepared from CuCN and 2 equiv of *n*-BuLi, at −78 °C for 2 h, followed by quenching the reaction with saturated aqueous NH_4_Cl, gave the corresponding carbocupration product **5** in 46% yield as a sole product (it is well known that the carbocupration reaction of fluoroalkylated alkynes with cuprates proceeds in a highly *cis*-selective manner [30–31]), together with a slight recovery of the starting material (Table 3, entry 1). In this case, the reaction proceeded in a highly regio- and stereoselective manner and the other isomers **6**–**12** were not detected at all (Figure 2). It was especially noteworthy that only the triple bond possessing a CF_3_ group, not the triple bond having a CMe_2_OBn group, was subjected to the carbocupration reaction. As shown in Table 3, entry 2, raising the reaction temperature from −78 to −45 °C led to a significant increase in the yield. We also examined the reaction with the cuprate prepared from Grignard reagent, *n*-BuMgBr. As summarised in Table 3, entries 3–5, a significant decrease of the yield was observed when the reaction was performed by using 1.2 equiv of cuprate. Very interestingly, the use of 1.5 equiv of the higher-ordered cyanocuprate realized the most satisfactory result, the desired product being obtained in 85% yield, though the yield was somewhat eroded in the reaction for 2 h.

**Table 3:** Investigation of the reaction conditions in carbocupration.

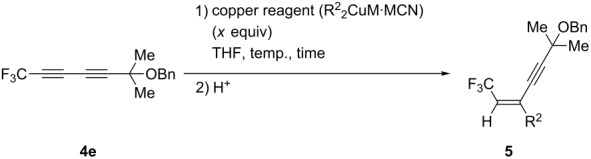						

entry	copper reagent^a^ (R^2^_2_CuM·MCN)	*x* (equiv)	temp. (°C)	time (h)	yield^b^ (% of **5)**	recovery^b^ (% of **4e)**

1	(*n*-Bu)_2_CuLi·LiCN	1.2	−78	2	46	7
2	(*n*-Bu)_2_CuLi·LiCN	1.2	−45	1	61	0
3	(*n*-Bu)_2_CuMgBr·MgBrCN	1.2	−45	1	38	0
4	(*n*-Bu)_2_CuMgBr·MgBrCN	1.2	−78	1	44	3
5	(*n*-Bu)_2_CuMgBr·MgBrCN	1.2	−78	2	44	0
6	(*n*-Bu)_2_CuMgBr·MgBrCN	1.5	−78	1	85	0
7	(*n*-Bu)_2_CuMgBr·MgBrCN	1.5	−78	2	57	0

^a^Copper reagents were prepared from 1 equiv of CuCN and 2 equiv of R^2^Li or R^2^MgBr. ^b^Determined by ^19^F NMR.

With the optimum reaction conditions in hand, we next investigated the carbocupration reaction by using various copper reagents. In all cases, iodine was employed as an electrophile instead of aqueous NH_4_Cl. The results are summarised in Table 4. As shown in Table 4, entries 1 and 2, (*n*-Bu)_2_CuLi·LiCN and Me_2_CuLi·LiCN could participate in the reaction to give the corresponding iodide **13a**,**b** in good yields. Furthermore, the cuprates prepared from alkyl Grignard reagents, such as *n*-Bu, Me, and cyclohexylmagnesium bromide, reacted smoothly (Table 4, entries 3–5). Switching the cuprate from dialkylcuprate into diarylcuprate did not bring about any influence on the yields at all (Table 4, entries 6 and 7).

**Table 4:** Carbocupration with various cuprates.

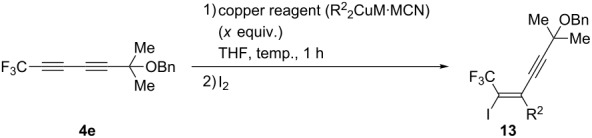					

entry	copper reagent^a^ (R^2^_2_CuM·MCN)	*x* (equiv)	temp. (°C)	product	yield^b^ (% of **13**)

1	(*n*-Bu)_2_CuLi·LiCN	1.2	−45	**13a**	54
2	Me_2_CuLi·LiCN	1.2	−45	**13b**	55
3	(*n*-Bu)_2_CuMgBr·MgBrCN	1.5	−78	**13a**	60
4	Me_2_CuMgBr·MgBrCN	1.5	−78	**13b**	59
5	Cy_2_CuMgBr·MgBrCN	1.5	−78	**13c**	44
6	Ph_2_CuMgBr·MgBrCN	1.5	−78	**13d**	55
7	(*p*-MeOC_6_H_4_)_2_CuMgBr·MgBrCN	1.5	−78	**13e**	49

^a^Copper reagents were prepared from 1 equiv of CuCN and 2 equiv of R^2^Li or R^2^MgBr. ^b^Isolated yield.

**Scheme 3:**
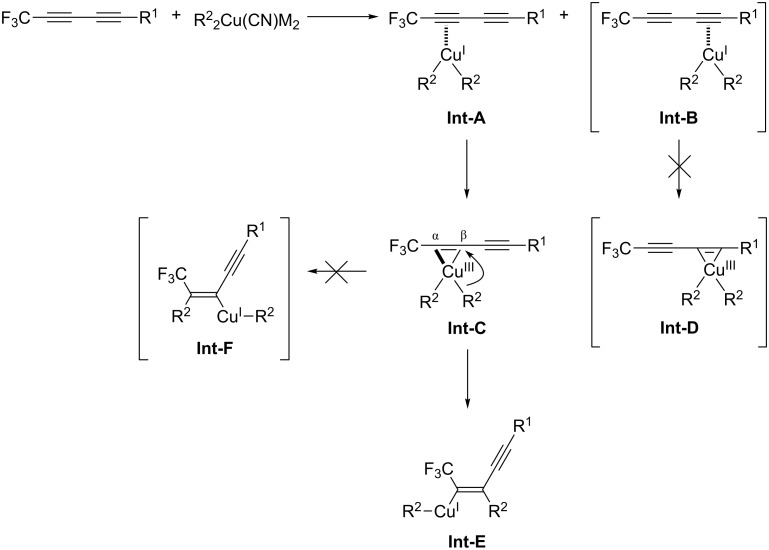
A proposed reaction mechanism.

A proposed reaction mechanism is outlined in Scheme 3. Based on the accumulated studies on the chemistry of fluoroalkylated alkynes, it appears possible that the copper reagent coordinates to the triple bond proximate to the CF_3_ group (**Int-A**), rather than the alternative one (**Int-B**), due to high reactivity of the fluoroalkylated alkyne. Then, Cu^I^ adds oxidatively to the alkyne to form the intermediate **Int-C,** not **Int-D**. Since a CF_3_ group has a very strong electron-withdrawing ability, the CF_3_C^α^—Cu^III^ bond may be stronger than Cu^III^—C^β^. (In the hydrometalation and the carbometalation reaction of fluoroalkylated alkynes, the same regioselectivity was observed [32–35].) Accordingly, a transfer of the R^2^ group on Cu^III^ to the olefinic carbon distal to a CF_3_ group may take place preferably, with vinylcopper intermediate **Int-E**, not **Int-F**, being produced exclusively. As a result, vinyl iodide **13** can be given in a highly regio- and stereoselective manner.

**Scheme 4:**
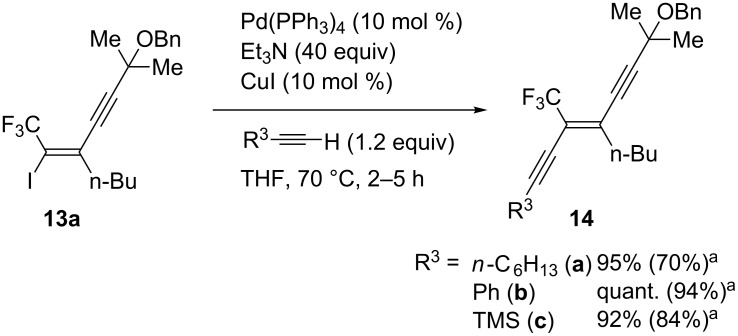
Synthesis of *trans*-enediynes. ^a^Determind by ^19^F NMR. Values in parentheses are of isolated yield.

Finally, we attempted the Sonogashira cross-coupling reaction of the obtained iodide **13a** (Scheme 4). Thus, treatment of **13a** with 1.2 equiv of terminal alkynes and 40 equiv of Et_3_N in the presence of 10 mol % each of Pd(PPh_3_)_4_ and CuI in THF at 70 °C for 2–5 h gave the corresponding enediynes **14a**–**c** in high to excellent yields. In all cases, other stereoisomers were not detected at all and **14a**–**c** were generated as the sole products in a pure form.

## Conclusion

In summary, we have established a convenient as well as efficient access to the trifluoromethylated diyne by Sonogashira cross-coupling reaction of readily accessible 2,3,3,3-tetrafluoro-1-iodo-1-propene (**1**) and the following HF elimination reaction. The thus-obtained CF_3_-enediyne could participate in the carbocupration with various higher-ordered cyanocuprates very well to give the corresponding vinyliodides in good yields. Finally, the thus-obtained iodide underwent a smooth Sonogashira cross-coupling reaction to afford the various desired *trans*-enediyne derivatives in high yields.

## Supporting Information

File 1 Experimental, characterization details, and NMR spectra of synthesized compounds, **4e**, **13a–e**, and **14a–c**.
