# Supplementary material for: Highly stereocontrolled synthesis of trans-enediynes via carbocupration of fluoroalkylated diynes
Source: Beilstein J Org Chem. 2012 Dec 19;8:2207–13. doi: 10.3762/bjoc.8.249 (PMC3558832; doi:10.3762/bjoc.8.249)

Supporting Information

for

Highly stereocontrolled synthesis of *trans*-enediynes via carbocupration of fluoroalkylated diynes

Tsutomu Konno*, Misato Kishi, and Takashi Ishihara

Address: Department of Chemistry and Materials Technology, Kyoto Institute of Technology, Matsugasaki, Sakyo-ku, Kyoto 606-0962, Japan

Email: Tsutomu Konno* – konno@chem.kit.ac.jp

* Corresponding author

Experimental, characterization details, and NMR spectra of synthesized compounds, 4e, 13a–e, and 14a–c

General methods

Infrared spectra (IR) were determined in a liquid film on a NaCl plate or KBr disk method with a JASCO FT/IR-4100 type A spectrometer. ^{1}H and ^{13}C NMR spectra were measured with a JEOL JNM-AL 400 NMR spectrometer in a chloroform-*d* ($CDCl_3$) solution with tetramethylsilane (Me_4Si) as an internal reference. A JEOL JNM-EX90A (84.21 MHz) FT-NMR spectrometer and a JEOL JNM-AL 400 NMR spectrometer were used for determining the yield of the products with hexafluorobenzene (C_6F_6). ^{19}F NMR (376.05 MHz) spectra were measured with a JEOL JNM-AL 400 NMR spectrometer in a chloroform-*d* ($CDCl_3$) solution with trichlorofluoromethane ($CFCl_3$) as an internal standard. High-resolution mass spectra (HRMS) were taken on a JEOL JMS-700MS spectrometer by electron impact (EI), chemical ionization (CI), and fast atom bombardment (FAB) methods.

All reactions were routinely monitored by ^{19}F NMR spectroscopy or TLC and carried out under an atmosphere of argon.

Materials

DMF, Et_3N and HMDS were fleshly distilled from calcium hydride (CaH_2). All chemicals were of reagent grade and, if necessary, were purified in the usual manner prior to use. Thin-layer chromatography (TLC) was done with Merck silica gel 60 F_{254} plates, and column chromatography was carried out using Wako gel C-200 as adsorbent.

Synthesis of 6-benzyloxy-1,1,1-trifluoro-6-methyl-2,4- heptadiyne (4e)

To a solution of HMDS (1.6 mL, 7.5 mmol) in THF (50 mL) was added 4.7 mL (7.5 mmol) of *n*-BuLi (1.6 M hexane solution) at -78 °C and the whole was stirred for 15 min. To this solution was added 6-benzyloxy-1,1,1,2-tetrafluoro-6-methyl-2-hepten-4-yne **3e** (1.43 g, 5.0 mmol) at -78 °C and the whole solution was stirred for 1 h. The reaction mixture was quenched with sat. NH_4Cl aq. and the whole was extracted with Et_2O three times. The combined organic layers were dried over anhydrous Na_2SO_4, then filtered. The filtrate was evaporated to give the crude materials, which was purified by silica gel column chromatography, affording the 6-benzyloxy-1,1,1-trifluoro-6-methyl-2,4-heptadiyne **4e** as yellow liquid (1.26 g, 4.74 mmol, 95% yield).

^{1}H NMR ($CDCl_3$) δ = 1.59 (s, 6H), 4.62 (s, 2H), 7.28–7.36 (m, 5H); ^{13}C NMR ($CDCl_3$) δ = 2.07, 28.16, 53.34, 64.04 (q, *J* = 53.71 Hz), 66.06 (q, *J* = 3.31 Hz), 66.63, 67.18, 70.70 (q, *J* = 6.63 Hz), 70.92, 87.24, 113.79 (q, *J* = 257.36 Hz), 127.67, 127.71, 128.41, 138.034; ^{19}F NMR ($CDCl_3$) δ = –50.38 (s, 3F); IR (neat) : 3033, 2989, 2868, 2266, 2171, 1467, 1383, 1321, 1149, 1054 cm^{-1}; HRMS (EI) calcd for $C_{15}H_{13}F_3O$ (M^+) 266.0918, found 266.0915.

Carbocupration of 6-benzyloxy-1,1,1-trifluoro-6-methyl-2,4-heptadiyne (4e)

To a solution of copper cyanide (54 mg, 0.6 mmol) in THF (2.0 mL) was added 0.75 mL (1.2 mmol) of *n*-BuLi (1.6 M hexane solution) at -45 °C and the whole was stirred for 10 min, then allowed to warm to -20 °C and stirred for 30 min. To this solution was added dropwise a solution of 6-benzyloxy-1,1,1-trifluoro-6-methyl-2,4-heptadiyne **4e** (133 mg, 0.5 mmol) in THF (0.5 mL). The reaction was stirred for 1 h at -45 °C, and was then quenched with NH_3 aq./MeOH (1 mL/5 mL) and extracted with Et_2O three times. The combined organic layers were dried over anhydrous Na_2SO_4 and concentrated *in vacuo*. The residue was chromatographed on silica gel to afford (*Z*)-6-benzyloxy-3-*n*-butyl-1,1,1-trifluoro-6-methyl-2-hepten-4-yne **5** (0.098 g, 0.30 mmol, 61% yield).

^{1}H NMR ($CDCl_3$) δ = 0.80–2.15 (m, 15H), 4.54 (s, 2H), 5.67 (q, *J* = 7.59 Hz, 1H), 7.17–7.28 (m, 5H); ^{13}C NMR ($CDCl_3$) δ = 13.78, 21.84, 22.64, 28.53 (q, *J* = 1.61 Hz), 29.75, 30.34, 37.48, 66.78 (t, *J* = 2.06 Hz), 71.04, 79.67 (q, *J* = 1.66 Hz), 101.68 (q, *J* = 1.71 Hz), 122.07 (qd, *J* = 33.89, 2.46 Hz), 122.36 (q, *J* = 270.01 Hz), 127.42, 127.79, 134.21 (q, *J* = 5.79 Hz), 138.82; ^{19}F NMR ($CDCl_3$) δ = –60.37 (d, *J* = 7.52 Hz, 3F); IR (neat) : 3066, 2934, 2864, 2224, 1642, 1497, 1455, 1379, 1123, 1056 cm^{-1}; HRMS (FAB) calcd for $C_{19}H_{23}F_3NaO$ (M+Na) 347.1599, found 347.1601.

Preparation of (*E*)-6-benzyloxy-3-*n*-butyl-1,1,1-trifluoro-2-iodo-6-methyl-2-hepten-4-yne (13a)

To a solution of copper cyanide (54 mg, 0.6 mmol) in THF (2.0 mL) was added 0.75 mL (1.2 mmol) of *n*-BuLi (1.6 M hexane solution) at -45 °C and the whole was stirred for 10 min, then allowed to warm to -20 °C and stirred for 30 min. To this solution was added dropwise a solution of 6-benzyloxy-1,1,1-trifluoro-6-methyl-2,4-heptadiyne **4e** (133 mg, 0.5 mmol) in THF (0.5 mL). The reaction was stirred for 1 h at -45 °C. After stirring at that temperature for 1 h, iodine (609 mg, 2.4 mmol) was added to the reaction mixture. After 1 h, the reaction was quenched with NH_3 aq./MeOH (1 mL/5 mL), extracted with Et_2O three times. The combined organic layers were dried over anhydrous Na_2SO_4 and concentrated *in vacuo*. The residue was chromatographed on silica gel to afford (*E*)-6-benzyloxy-3-*n*-butyl-1,1,1-trifluoro-2-iodo-6-methyl-2-hepten-4-yne **13a** (0.121 g, 0.27 mmol, 54% yield).

^{1}H NMR ($CDCl_3$) δ = 0.92–2.51 (m, 15H), 4.61 (s, 2H), 7.26–7.36 (m, 5H); ^{13}C NMR ($CDCl_3$) δ = 2.10, 13.87, 22.06, 28.38, 28.39, 29.37, 43.08, 66.84 (t, *J* = 2.05 Hz), 71.12, 79.22, 93.75 (q, *J* = 34.44 Hz), 102.69 (q, *J* = 2.21 Hz), 120.77 (q, *J* = 2.21 Hz), 127.45, 127.71, 128.31, 138.08 (q, *J* = 3.58 Hz), 138.69, 140.70; ^{19}F NMR ($CDCl_3$) δ = –57.12 (s, 3F); IR (neat) : 3090, 3032, 2960, 2933, 2864, 2220, 1947, 1806, 1710, 1643, 1577, 1455, 1380, 1240, 1159, 1057 cm^{-1}; HRMS (FAB) calcd for $C_{19}H_{22}F_3INaO$ (M+Na) 473.0565, found 473.0565.

(*E*)-6-Benzyloxy-1,1,1-trifluoro-2-iodo-3,6-dimethyl-2-hepten-4-yne (13b)

^{1}H NMR ($CDCl_3$) δ = 1.57 (s, 6H), 2.22 (s, 3H), 4.60 (s, 2H), 7.25–7.36 (m, 5H); ^{13}C NMR ($CDCl_3$) δ = 28.36, 30.98 (q, *J* = 1.64 Hz), 66.80 (t, *J* = 2.06 Hz), 71.07, 80.11, 94.47 (q, *J* = 34.70 Hz), 102.18, 102.20, 120.77 (q, *J* = 273.02 Hz), 127.46, 127.72, 128.32, 128.99, 129.76, 133.67 (q, *J* = 3.58 Hz), 134.48, 138.72; ^{19}F NMR ($CDCl_3$) δ = –57.50 (s, 3F); IR (neat): 3033, 2986, 1703, 1454, 1296, 1241, 1131 , 1056 cm^{-1}; HRMS (FAB) calcd for $C_{16}H_{16}F_3INaO$ (M+Na) 431.0096, found 431.0087.

(*E*)-6-Benzyloxy-3-cyclohexyl-1,1,1-trifluoro-2-iodo-6-methyl-2-hepten-4-yne (13c)

^{1}H NMR ($CDCl_3$) δ = 1.32–1.83 (m, 17H), 4.65 (s, 2H), 7.26–7.40 (m, 5H); ^{13}C NMR ($CDCl_3$) δ = 2.11, 25.59, 25.72, 25.89, 31.42, 28.45, 28.47, 28.58, 30.21, 45.71, 50.86, 66.95 (t, *J* = 2,51 Hz), 71.11, 71.24, 92.72 (q, *J* = 34.17 Hz), 103.72 (q, *J* = 2.48 Hz), 120.83 (q, *J* = 273.59 Hz), 127.48, 127.75, 128.33, 142.25 (q, *J* = 3.31 Hz); ^{19}F NMR ($CDCl_3$) δ = –56.45 (s, 3F); IR (neat): 3584, 3032, 2932, 2856, 1569, 1451, 1288, 1129, 1056 cm^{-1}; HRMS (FAB) calcd for $C_{21}H_{24}F_3INaO$ (M+Na) 499.0722, found 499.0723.

(*E*)-6-Benzyloxy-1,1,1-trifluoro-2-iodo-6-methyl-3-phenyl-2-hepten-4-yne (13d)

^{1}H NMR ($CDCl_3$) δ = 1.56 (s, 6H), 4.56 (s, 2H), 7.26–7.43 (m, 10H); ^{13}C NMR ($CDCl_3$) δ = 28.25, 28.53, 66.90 (t, *J* = 1.66 Hz), 71.19, 80.11, 94.91 (q, *J* = 34.44 Hz), 104.68 (q, *J* = 2.21 Hz), 121.11 (q, *J* = 273.29 Hz), 127.45, 127.82, 128.29, 128.53, 129.04, 130.00, 130.40, 138.01 (q, *J* = 3.85 Hz), 138.58, 138.70, 141.73; ^{19}F NMR ($CDCl_3$) δ = –57.74 (s, 3F); IR (neat): 3584, 2985, 1563, 1454, 1299, 1248, 1132, 1053 cm^{-1}; HRMS (FAB) calcd for $C_{21}H_{18}F_3INaO$ (M+Na) 493.0252, found 493.0251.

(*E*)-6-Benzyloxy-1,1,1-trifluoro-2-iodo-6-methyl-3-*p*-methoxyphenyl-2-hepten-4-yne (13e)

^{1}H NMR ($CDCl_3$) δ = 1.59 (s, 6H), 3.86 (s, 3H), 4.59 (s, 2H), 6.93–7.52 (m, 9H); ^{13}C NMR ($CDCl_3$) δ = 28.26, 55.26, 55.28, 66.87 (t, *J* = 2.06 Hz), 71.19, 80.32, 93.67 (q, *J* = 34.17 Hz), 104.21 (q, *J* = 2.48 Hz), 113.67, 114.12, 121.18 (q, *J* = 273.59 Hz), 127.43, 127.69, 127.80, 128.27, 129.62, 133.42, 133.70, 137.59 (q, *J* = 3.58 Hz), 138.62, 158.64, 160.07; ^{19}F NMR ($CDCl_3$) δ = –57.35 (s, 3F); IR (neat) : 3584, 3033, 2985, 2936, 2839, 2212, 1888, 1606, 1508, 1249, 1130, 1041 cm^{-1}; HRMS (FAB) calcd for $C_{22}H_{20}F_3IO_2$ (M+) 500.0460, found 500.0463.

Typical procedure for the preparation of 2-benzyloxy-2-methyl-6-trifluoromethyl-5-tetradecen-3,7-diyne (14a)

To a solution of (*E*)-6-benzyloxy-3-*n*-butyl-1,1,1-trifluoro-2-iodo-6-methyl-2-hepten-4-yne **13a** (0.450 g, 1.0 mmol), 1-octyne (0.220 g, 2.0 mmol), CuI (0.019 g. 0.10 mmol) in THF (10 mL) was

added $Pd(PPh_3)_4$ (0.116 mg, 0.10 mmol) followed by Et_3N (5.6 mL, 40 mmol) at room temperature. The whole was allowed to warm to 70 °C, then stirred 2 h, and then quenched with sat. NH_4Cl aq. The mixture was extracted with Et_2O three times, and the combined organic layers were dried over anhydrous Na_2SO_4, and then filtered. The filtrate was evaporated to give the corresponding crude materials, which were purified by silica-gel column chromatography, affording the (*Z*)-2-benzyloxy-6-*n*-butyl-2-methyl-6-trifluoromethyl-5-pentadecen-3,7-diyne **14a** (0.301 g, 0.70 mmol, 70% yield.)

^{1}H NMR ($CDCl_3$) δ = 0.88–2.53 (m, 28H), 4.62 (s, 2H), 7.29–7.38 (m, 5H); ^{13}C NMR ($CDCl_3$) δ = 13.90 (d, *J* = 12.35 Hz), 19.77, 22.29 (d, *J* = 43.87 Hz), 28.26, 28.46 (d, *J* = 5.82 Hz), 29.81, 31.24, 35.86, 66.82 (t, *J* = 2.05 Hz), 71.23, 73.60 (q, *J* = 2.51 Hz), 80.99, 103.50, 105.54 (q, *J* = 1.61 Hz), 119.23 (q, *J* = 33.03 Hz), 121.53 (q, *J* = 274.43 Hz), 127.39, 127.76, 128.27, 128.42, 128.48, 128.67, 133.70 (d, *J* = 18.98 Hz), 136.15 (q, *J* = 2.48 Hz), 137.13 (d, *J* = 1.71 Hz), 137.23, 138.83; ^{19}F NMR ($CDCl_3$) δ = –61.58 (s, 3F); IR (neat) : 3032, 2958, 2932, 2861, 2222, 1585, 1347, 1160, 1131, 1057 cm^{-1}; HRMS (FAB) calcd for $C_{27}H_{35}F_3NaO$ (M+Na) 455.2538, found 455.2542.

(*Z*)-7-Benzyloxy-4-*n*-butyl-7-methyl-1-phenyl-3-trifluoromethyl-3-octen-1,5-diyne (14b)

^{1}H NMR ($CDCl_3$) δ = 0.91–2.63 (m, 15H), 4.64 (s, 2H), 7.26–7.48 (m, 10H); ^{13}C NMR ($CDCl_3$) δ = 13.89, 15.26, 22.11, 28.49, 29.90, 36.17, 65.85, 66.89 (t, *J* = 2.46 Hz), 71.28, 81.10, 82.07 (q, *J* = 2.48 Hz), 101.43, 106.81 (q, *J* = 1.91 Hz), 118.98 (q, *J* = 33.63 Hz), 121.43 (q, *J* = 274.43 Hz), 122.26, 127.45, 127.79, 128.32, 128.45, 129.09, 131.45, 137.60 (q, *J* = 2.48 Hz), 138.76; ^{19}F NMR ($CDCl_3$) δ = –61.27 (s, 3F); IR (neat): 3033, 2959, 2932, 2862, 2214, 1576, 1491, 1355, 1235, 1133, 1058, 1000 cm^{-1}; HRMS (FAB) calcd for $C_{27}H_{27}F_3NaO$ (M+Na) 447.1912, found 447.1909.

(*Z*)-7-Benzyloxy-4-*n*-butyl-7-methyl-3-trifluoromethyl-1-trimethylsilyl-3-octen-1,5-diyne (14c)

^{1}H NMR ($CDCl_3$) δ = 0.25 (s, 9H), 0.93–1.63 (m, 13H), 7.19 (t, *J* = 7.19 Hz, 2H), 4.63 (s, 2H), 7.26–7.38 (m, 5H); ^{13}C NMR ($CDCl_3$) δ = –0.40, 13.80, 22.04, 28.44, 29.71, 36.09, 43.11, 66.87 (t, *J* = 2.06 Hz), 71.25, 80.89, 96.61 (q, *J* = 2.48 Hz), 107.15 (q, *J* = 1.67 Hz), 108.19, 118.97 (q, *J* = 33.60 Hz), 121.22 (q, *J* = 274.16 Hz), 127.45, 127.78, 128.31, 129.26, 138.74, 139.11 (q, *J* = 2.74 Hz); ^{19}F NMR ($CDCl_3$) δ = –61.38 (s, 3F); IR (neat): 3033, 2960, 2933, 2863, 2144, 1578, 1455, 1339, 1253, 1134, 1057 cm^{-1}; HRMS (FAB) calcd for $C_{24}H_{31}F_3NaOSi$ (M+Na) 443.1994, found 443.1992.

auto

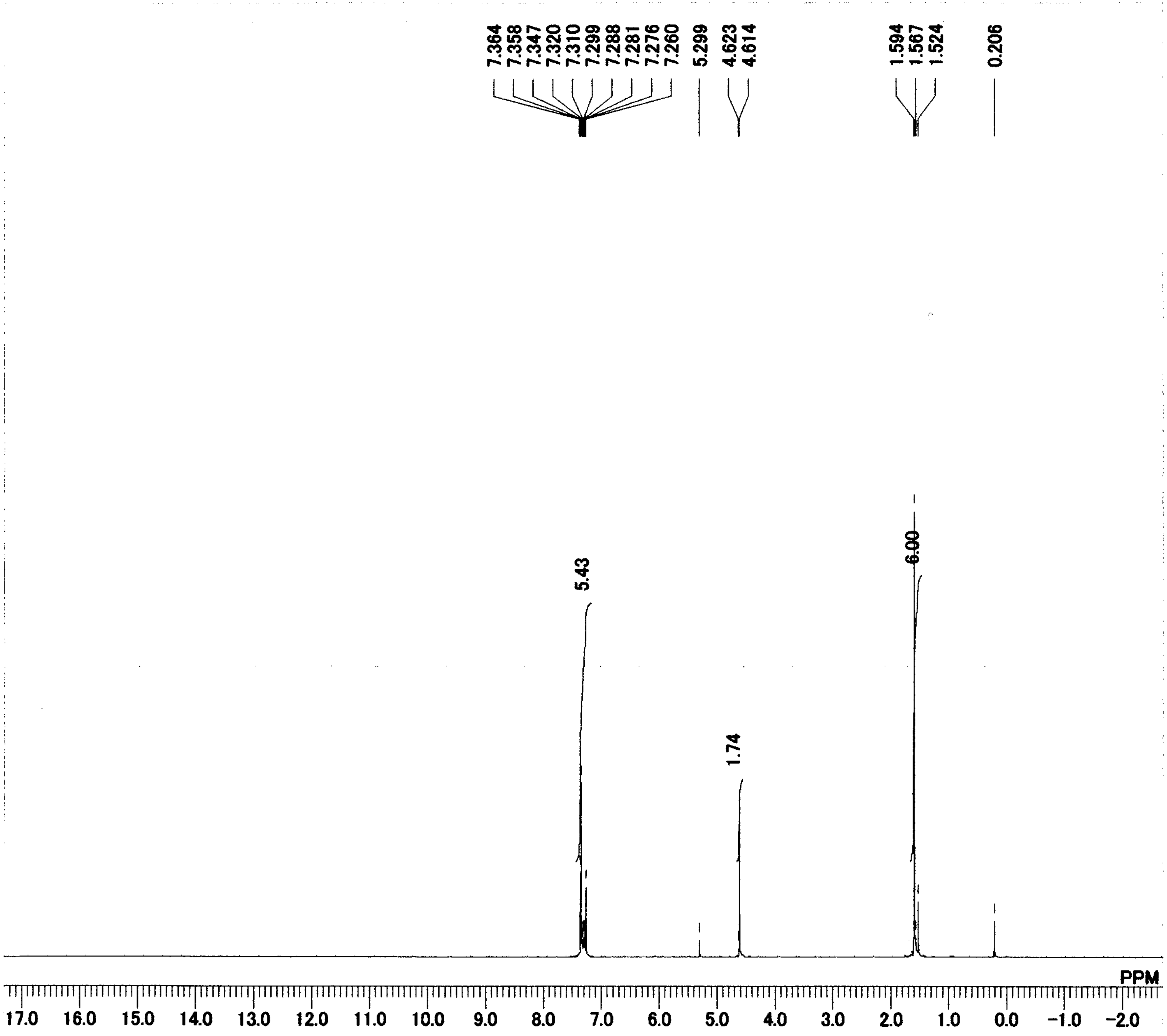

MENUF	1H
OBNUC	1H
OFR	399.65 MHz
OBSET	135.40 KHz
OBFIN	24.90 Hz
PW1	5.80 usec
DEADT	72.10 usec
PREDL	0.20000 msec
IWT	1.0000 msec
POINT	16384
SPO	16384
TIMES	8
DUMMY	1
FREQU	7992.01 Hz
FLT	4000 Hz
DELAY	50.00 usec
ACQTM	2.0501 sec
PD	4.9500 sec
ADBIT	16
RGAIN	21
BF	0.60 Hz
T1	0.00
T2	0.00
T3	90.00
T4	100.00
EXMOD	NON
EXPCM	NON:Single.coupled:PW1_ACQTM_PI
IRNUC	1H
IFR	399.65 MHz
IRSET	136.90 KHz
IRFIN	97.50 Hz
IRRPW	45 usec
IRATN	511
DFILE	Rxn273 カラム H.als
SF	TH5ATFG2
LKSET	61.60 KHz
LKFIN	79.0 Hz
LKLEV	180
LGAIN	24
LKPHS	250
LKSIG	1048
CSPED	13 Hz
FILDC	
FILDF	

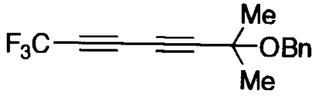

auto

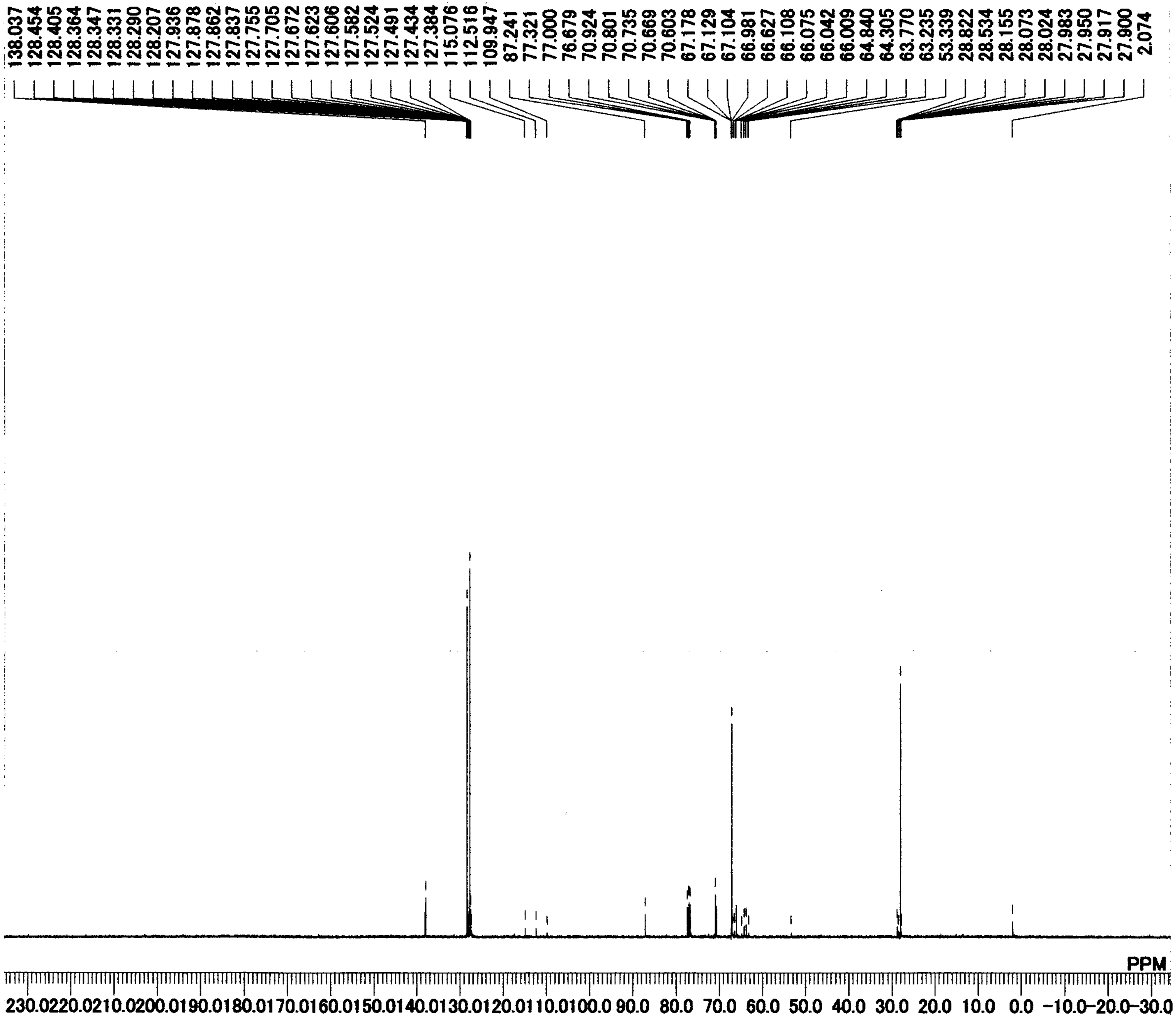

MENUF	13C
OBNUC	13C
OFR	100.40 MHz
OBSET	125.00 KHz
OBFIN	10500.00 Hz
PW1	6.00 usec
DEADT	19.10 usec
PREDL	0.20000 msec
IWT	1.0000 msec
POINT	32768
SPO	32768
TIMES	512
DUMMY	1
FREQU	27118.64 Hz
FLT	13550 Hz
DELAY	14.80 usec
ACQTM	1.2083 sec
PD	1.7920 sec
ADBIT	16
RGAIN	25
BF	0.10 Hz
T1	0.00
T2	0.00
T3	90.00
T4	100.00
EXMOD	BCM
EXPCM	Bilevel.complete.decoupling;Set_IRRF
IRNUC	1H
IFR	399.65 MHz
IRSET	124.00 KHz
IRFIN	10500.00 Hz
IRRPW	45 usec
IRATN	511
DFILE	Rxn273 ジイン C.als
SF	TH5ATFG2
LKSET	61.60 KHz
LKFIN	79.0 Hz
LKLEV	180
LGAIN	23
LKPHS	250
LKSIG	726
CSPED	10 Hz
FILDC	
FILDF	

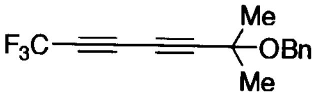

auto

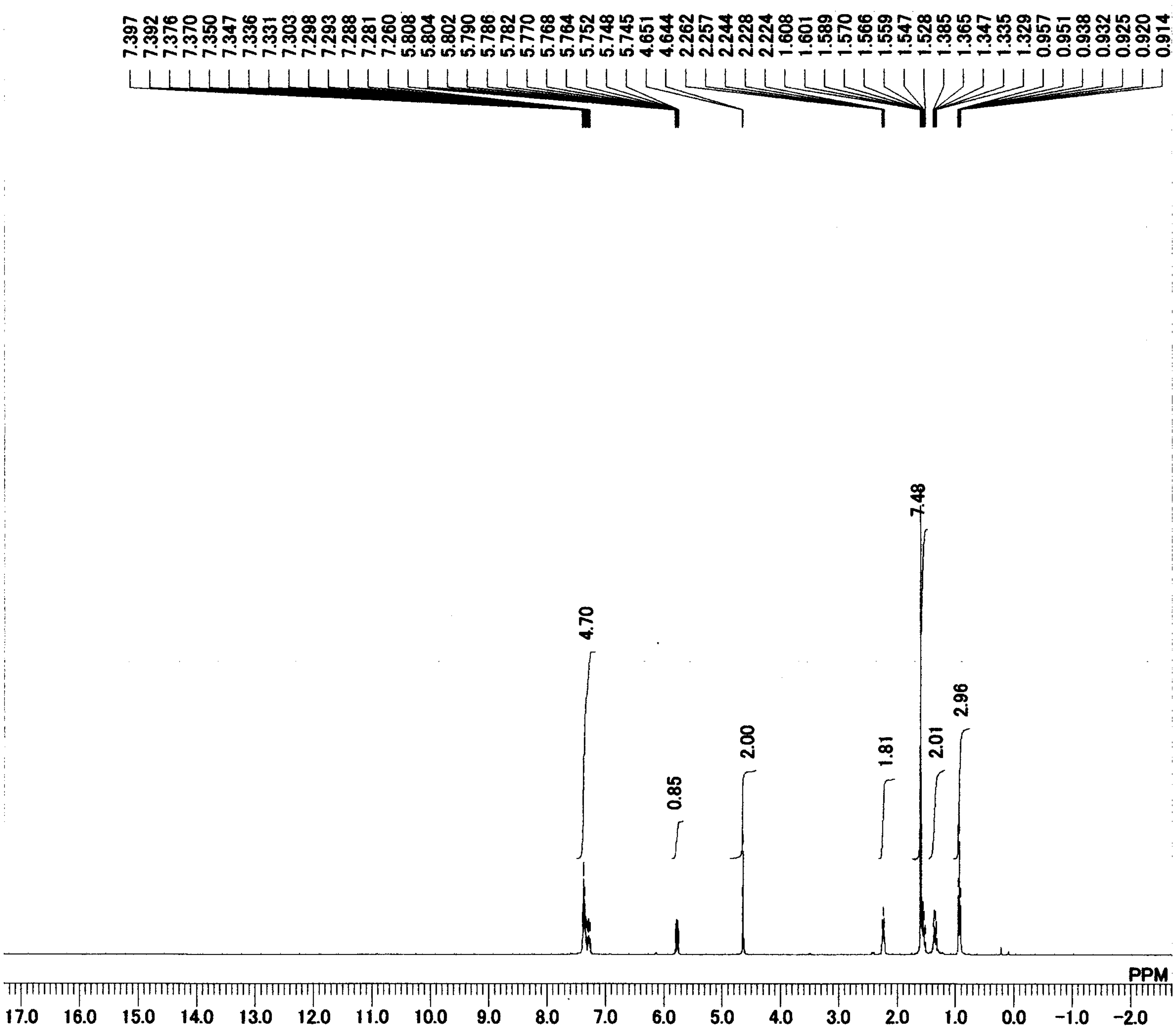

MENUF	1H
OBNUC	1H
OFR	399.65 MHz
OBSET	135.40 KHz
OBFIN	24.90 Hz
PW1	5.50 usec
DEADT	72.20 usec
PREDL	0.20000 msec
IWT	1.0000 msec
POINT	16384
SPO	16384
TIMES	8
DUMMY	1
FREQU	7992.01 Hz
FLT	4000 Hz
DELAY	50.00 usec
ACQTM	2.0501 sec
PD	4.9500 sec
ADBIT	16
RGAIN	11
BF	0.10 Hz
T1	0.00
T2	0.00
T3	90.00
T4	100.00
EXMOD	NON
EXPCM	NON:Single.coupled:PW1_ACQTM_PE
IRNUC	1H
IFR	399.65 MHz
IRSET	136.90 KHz
IRFIN	97.50 Hz
IRRPW	45 usec
IRATN	511
DFILE	Rxn464 再カラム 1H.als
SF	TH5ATFG2
LKSET	61.60 KHz
LKFIN	79.0 Hz
LKLEV	180
LGAIN	23
LKPHS	240
LKSIG	897
CSPED	11 Hz
FILDC	
FILDF	

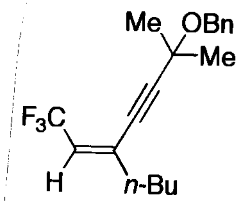

auto

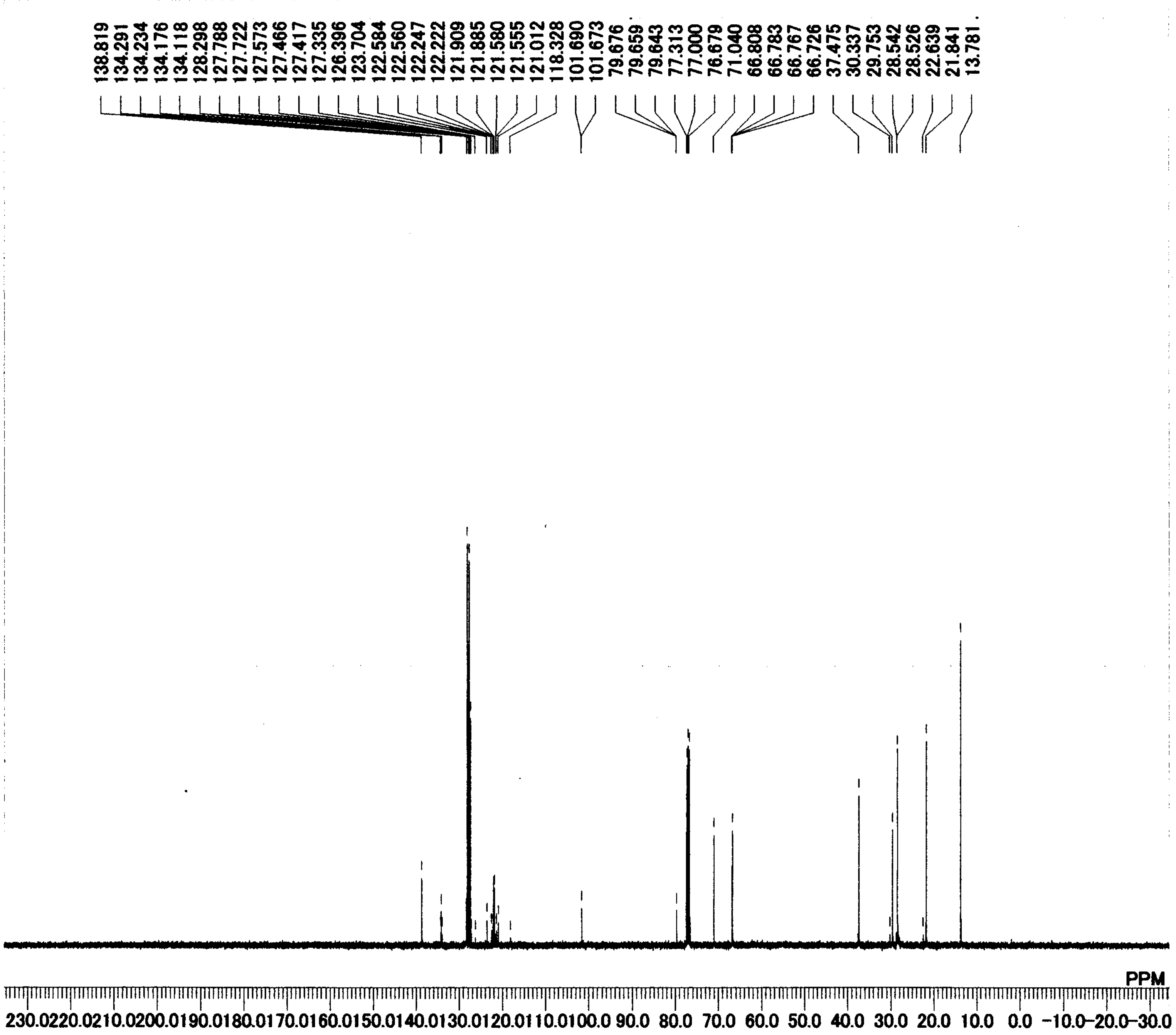

MENUF	13C
OBNUC	13C
OFR	100.40 MHz
OBSET	125.00 KHz
OBFIN	10500.00 Hz
PW1	6.20 usec
DEADT	19.00 usec
PREDL	0.20000 msec
IWT	1.0000 msec
POINT	32768
SPO	32768
TIMES	800
DUMMY	1
FREQU	27118.64 Hz
FLT	13550 Hz
DELAY	14.80 usec
ACQTM	1.2083 sec
PD	1.7920 sec
ADBIT	16
RGAIN	25
BF	0.10 Hz
T1	0.00
T2	0.00
T3	90.00
T4	100.00
EXMOD	BCM
EXPCM	Bilevel.complete.decoupling:Set_IRRF
IRNUC	1H
IFR	399.65 MHz
IRSET	124.00 KHz
IRFIN	10500.00 Hz
IRRPW	45 usec
IRATN	511
DFILE	Rxn464 Cu→H C.als
SF	TH5ATFG2
LKSET	61.60 KHz
LKFIN	79.0 Hz
LKLEV	180
LGAIN	23
LKPHS	240
LKSIG	853
CSPED	11 Hz
FILDC	
FILDF	

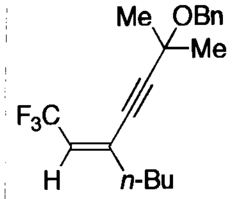

auto

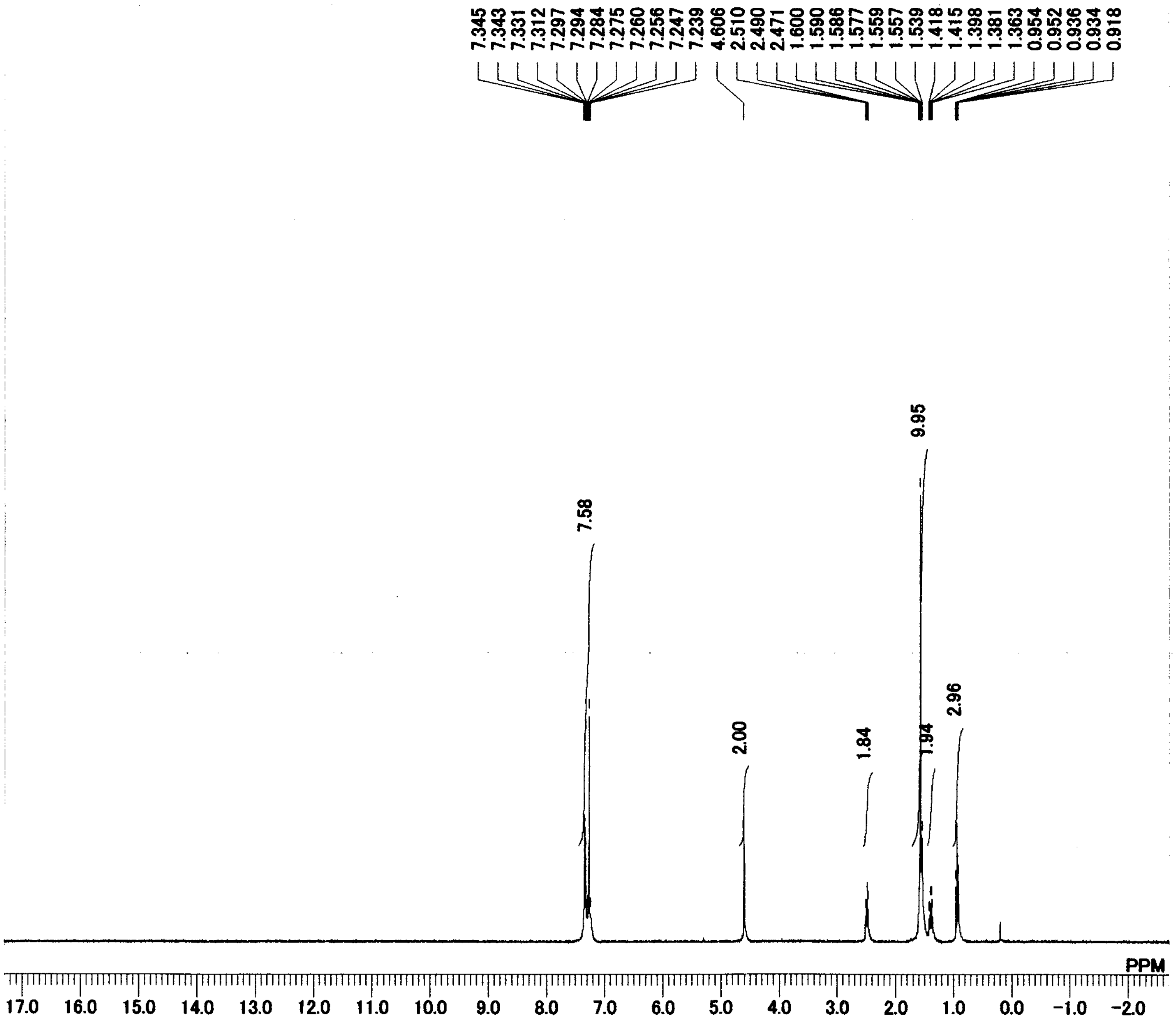

MENUF	1H
OBNUC	1H
OFR	399.65 MHz
OBSET	135.40 KHz
OBFIN	24.90 Hz
PW1	5.80 usec
DEADT	72.10 usec
PREDL	0.20000 msec
IWT	1.0000 msec
POINT	16384
SPO	16384
TIMES	8
DUMMY	1
FREQU	7992.01 Hz
FLT	4000 Hz
DELAY	50.00 usec
ACQTM	2.0501 sec
PD	4.9500 sec
ADBIT	16
RGAIN	20
BF	0.00 Hz
T1	0.00
T2	0.00
T3	90.00
T4	100.00
EXMOD	NON
EXPCM	NON:Single.coupled:PW1_ACQTM_PD
IRNUC	1H
IFR	399.65 MHz
IRSET	136.90 KHz
IRFIN	97.50 Hz
IRRPW	45 usec
IRATN	511
DFILE	Rxn376 カラム H.als
SF	TH5ATFG2
LKSET	61.60 KHz
LKFIN	79.0 Hz
LKLEV	180
LGAIN	25
LKPHS	193
LKSIG	683
CSPED	12 Hz
FILDC	
FILDF	

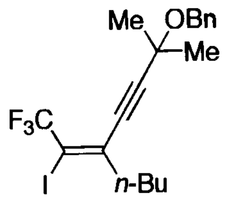

auto

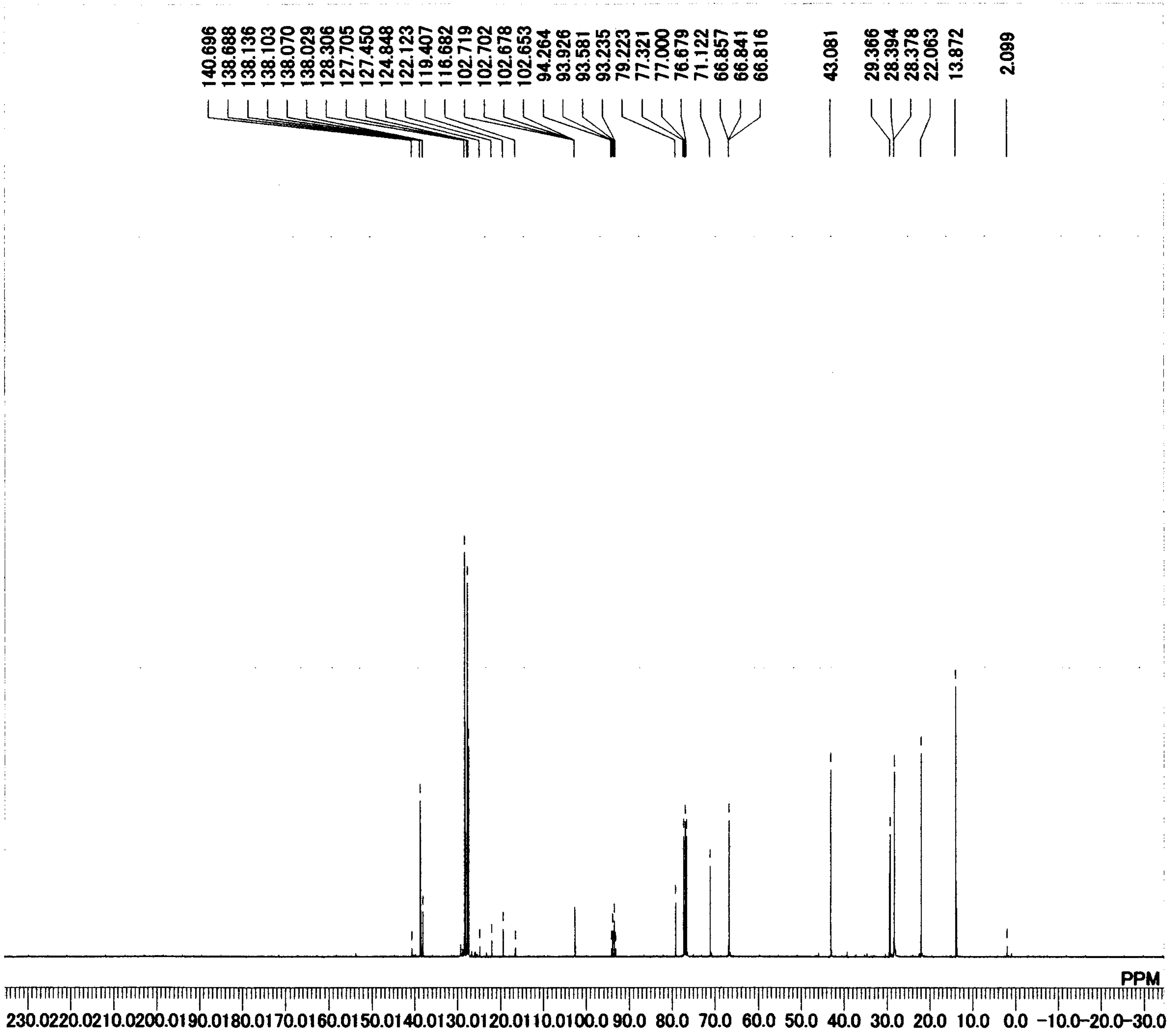

MENUF	13C
OBNUC	13C
OFR	100.40 MHz
OBSET	125.00 KHz
OBFIN	10500.00 Hz
PW1	6.20 usec
DEADT	19.00 usec
PREDL	0.20000 msec
IWT	1.0000 msec
POINT	32768
SPO	32768
TIMES	20000
DUMMY	1
FREQU	27118.64 Hz
FLT	13550 Hz
DELAY	14.80 usec
ACQTM	1.2083 sec
PD	1.7920 sec
ADBIT	16
RGAIN	25
BF	0.10 Hz
T1	0.00
T2	0.00
T3	90.00
T4	100.00
EXMOD	BCM
EXPCM	Bilevel.complete.decoupling:Set_IRRF
IRNUC	1H
IFR	399.65 MHz
IRSET	124.00 KHz
IRFIN	10500.00 Hz
IRRPW	45 usec
IRATN	511
DFILE	Rxn469 Cu→I C.als
SF	TH5ATFG2
LKSET	61.60 KHz
LKFIN	79.0 Hz
LKLEV	180
LGAIN	24
LKPHS	240
LKSIG	985
CSPED	10 Hz
FILDC	
FILDF	

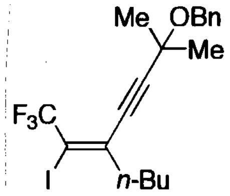

auto

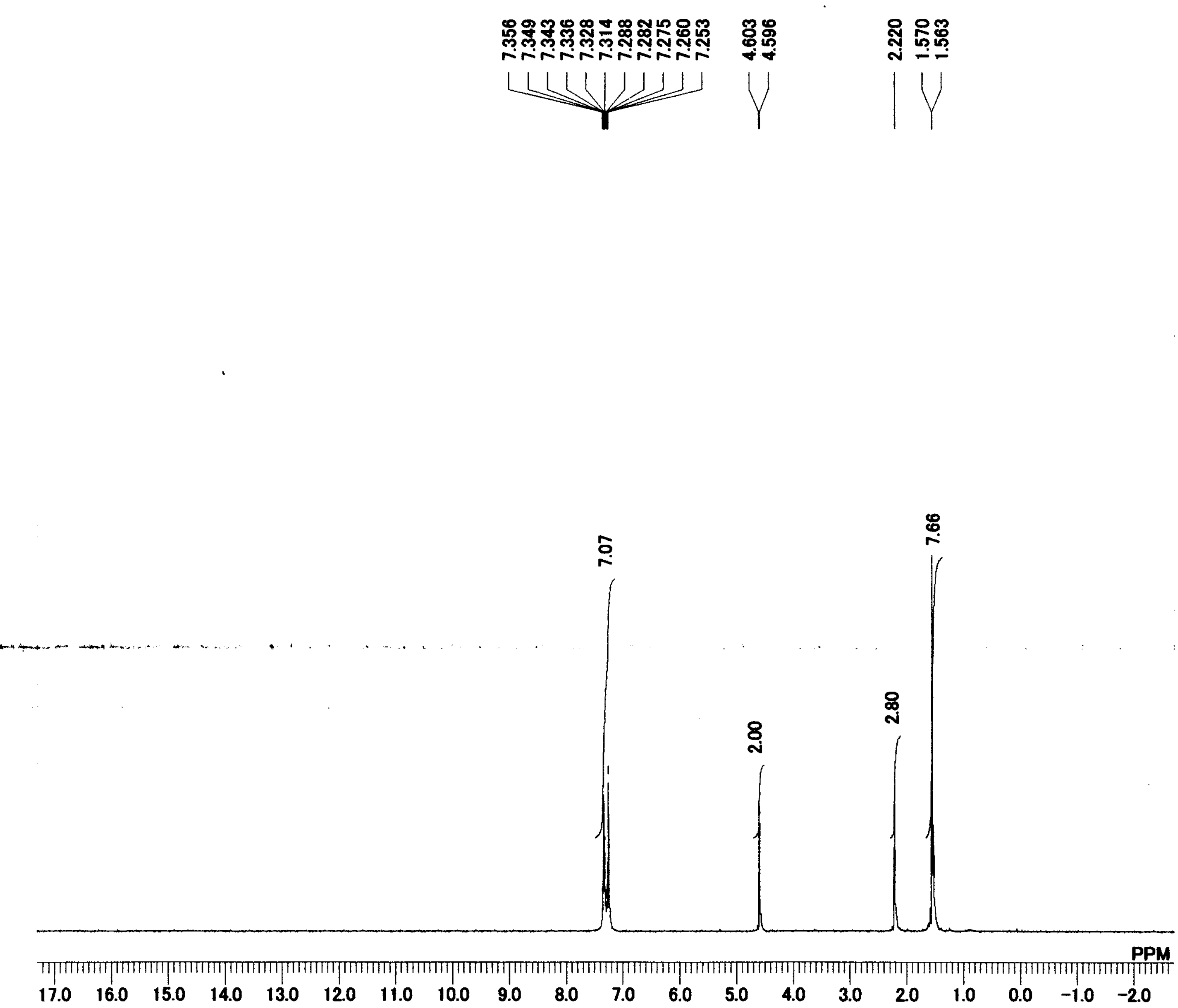

MENUF	1H
OBNUC	1H
OFR	399.65 MHz
OBSET	135.40 KHz
OBFIN	24.90 Hz
PW1	5.80 usec
DEADT	72.10 usec
PREDL	0.20000 msec
IWT	1.0000 msec
POINT	16384
SPO	16384
TIMES	8
DUMMY	1
FREQU	7992.01 Hz
FLT	4000 Hz
DELAY	50.00 usec
ACQTM	2.0501 sec
PD	4.9500 sec
ADBIT	16
RGAIN	21
BF	0.10 Hz
T1	0.00
T2	0.00
T3	90.00
T4	100.00
EXMOD	NON
EXPCM	NON:Single.coupled:PW1_ACQTM_PI
IRNUC	1H
IFR	399.65 MHz
IRSET	136.90 KHz
IRFIN	97.50 Hz
IRRPW	45 usec
IRATN	511
DFILE	悪.als
SF	TH5ATFG2
LKSET	61.60 KHz
LKFIN	79.0 Hz
LKLEV	180
LGAIN	25
LKPHS	193
LKSIG	679
CSPED	11 Hz
FILDC	
FILDF	

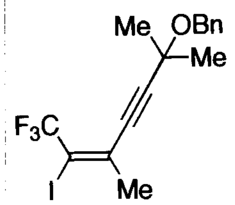

auto

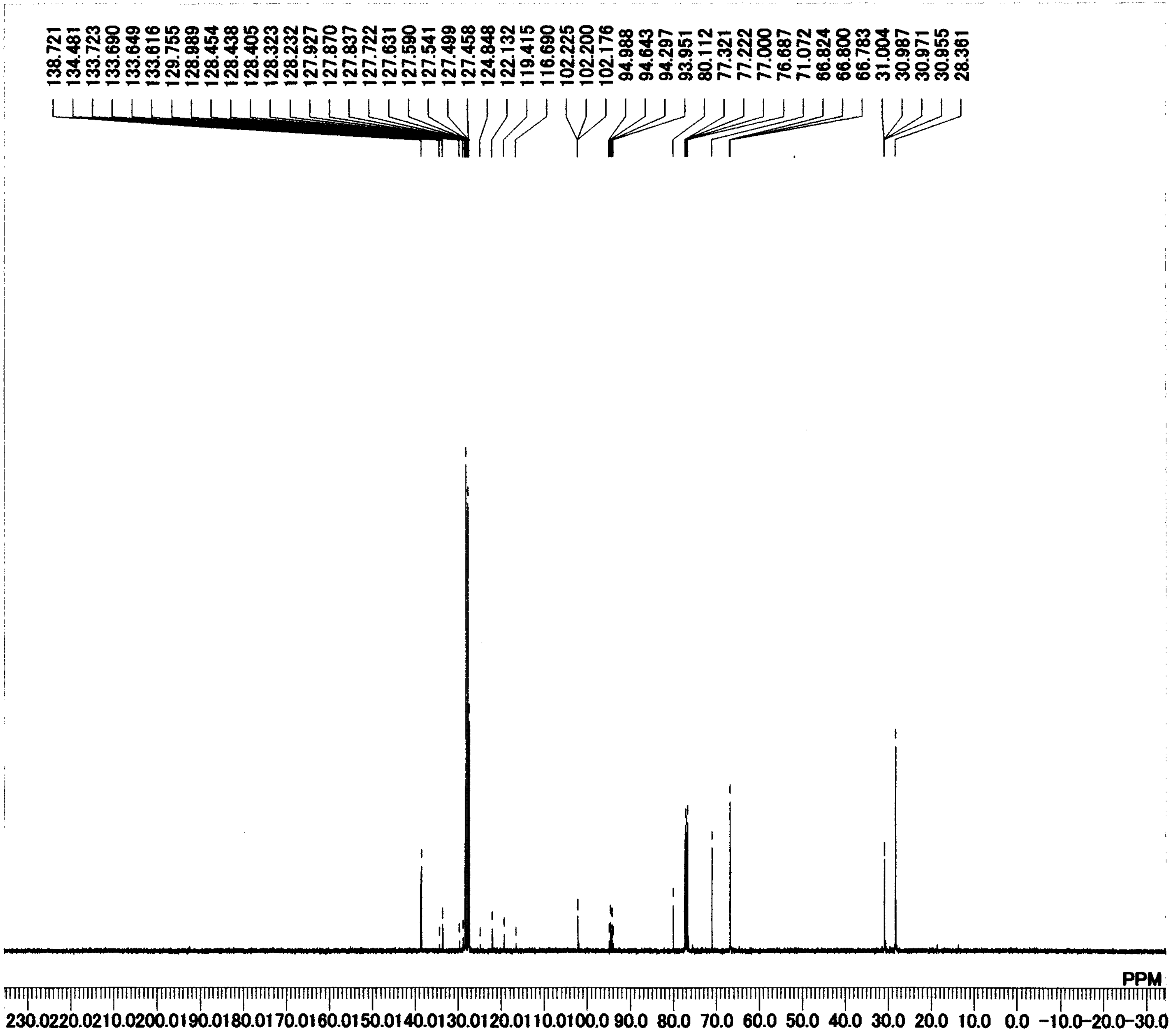

MENUF	13C
OBNUC	13C
OFR	100.40 MHz
OBSET	125.00 KHz
OBFIN	10500.00 Hz
PW1	6.20 usec
DEADT	19.00 usec
PREDL	0.20000 msec
IWT	1.0000 msec
POINT	32768
SPO	32768
TIMES	2000
DUMMY	1
FREQU	27118.64 Hz
FLT	13550 Hz
DELAY	14.80 usec
ACQTM	1.2083 sec
PD	1.7920 sec
ADBIT	16
RGAIN	25
BF	0.10 Hz
T1	0.00
T2	0.00
T3	90.00
T4	100.00
EXMOD	BCM
EXPCM	Bilevel.complete.decoupling:Set_IRRF
IRNUC	1H
IFR	399.65 MHz
IRSET	124.00 KHz
IRFIN	10500.00 Hz
IRRPW	45 usec
IRATN	511
DFILE	Rxn377 carbocup Me C.als
SF	TH5ATFG2
LKSET	61.60 KHz
LKFIN	79.0 Hz
LKLEV	180
LGAIN	23
LKPHS	240
LKSIG	794
CSPED	11 Hz
FILDC	
FILDF	

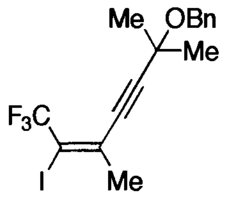

auto

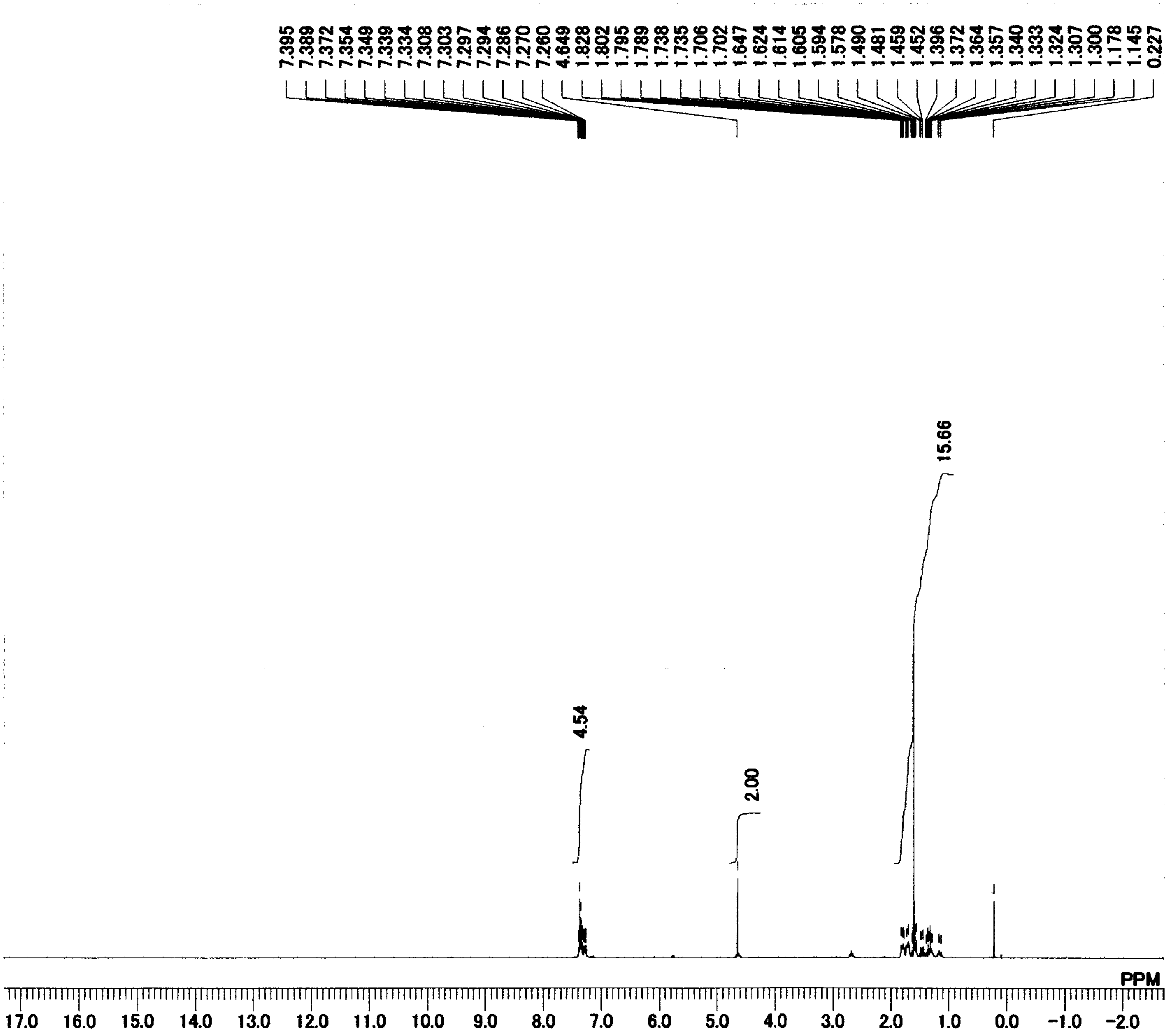

MENUF	1H
OBNUC	1H
OFR	399.65 MHz
OBSET	135.40 KHz
OBFIN	24.90 Hz
PW1	5.50 usec
DEADT	72.20 usec
PREDL	0.20000 msec
IWT	1.0000 msec
POINT	16384
SPO	16384
TIMES	8
DUMMY	1
FREQU	7992.01 Hz
FLT	4000 Hz
DELAY	50.00 usec
ACQTM	2.0501 sec
PD	4.9500 sec
ADBIT	16
RGAIN	11
BF	0.10 Hz
T1	0.00
T2	0.00
T3	90.00
T4	100.00
EXMOD	NON
EXPCM	NON:Single.coupled:PW1_ACQTM_PD
IRNUC	1H
IFR	399.65 MHz
IRSET	136.90 KHz
IRFIN	97.50 Hz
IRRPW	45 usec
IRATN	511
DFILE	Rxn481 c-Hex カラム H.als
SF	TH5ATFG2
LKSET	61.60 KHz
LKFIN	79.0 Hz
LKLEV	180
LGAIN	25
LKPHS	240
LKSIG	1749
CSPED	14 Hz
FILDC	
FILDF	

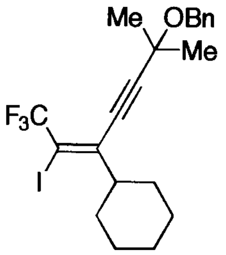

auto

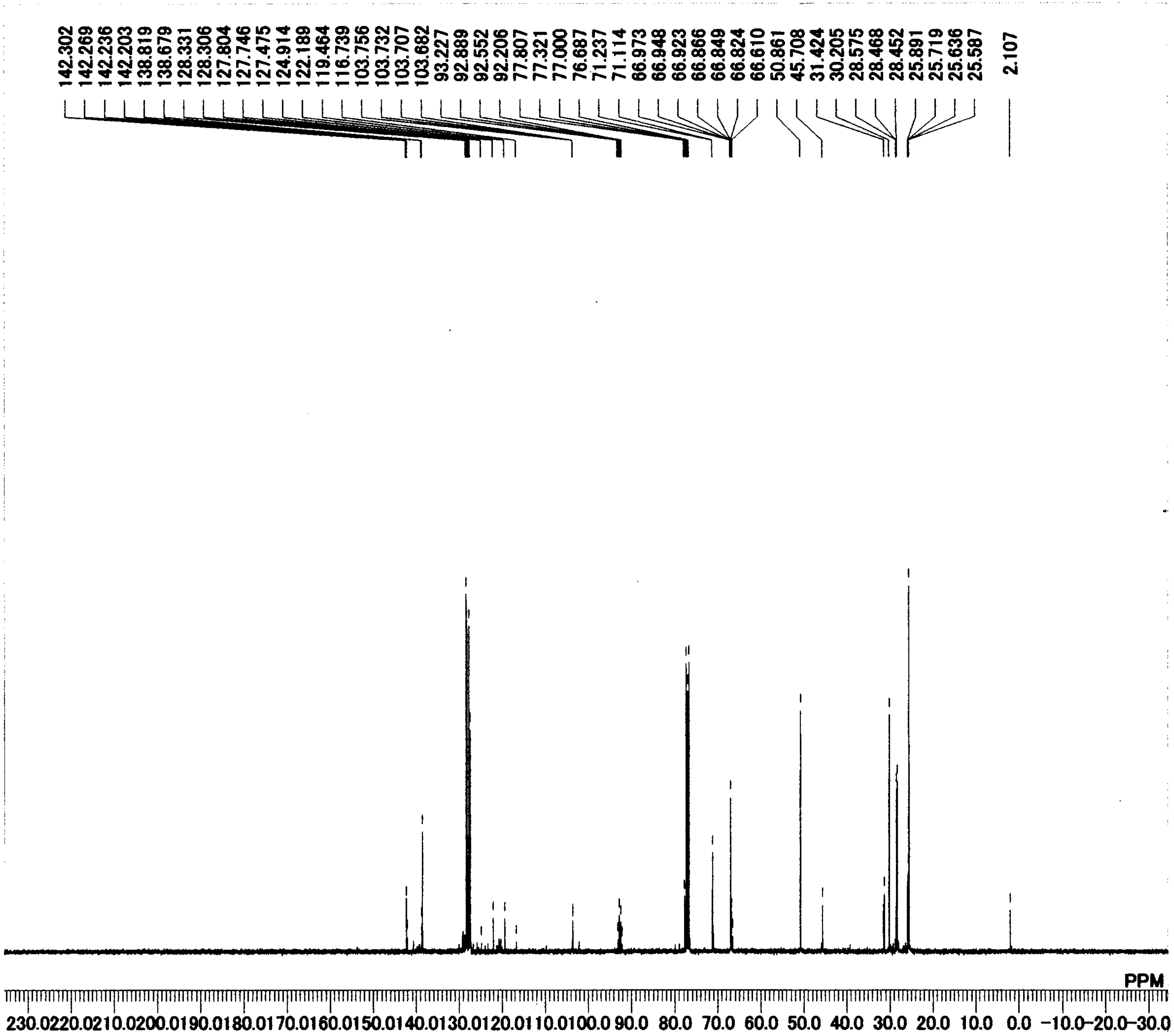

MENUF	13C
OBNUC	13C
OFR	100.40 MHz
OBSET	125.00 KHz
OBFIN	10500.00 Hz
PW1	6.20 usec
DEADT	19.00 usec
PREDL	0.20000 msec
IWT	1.0000 msec
POINT	32768
SPO	32768
TIMES	10000
DUMMY	1
FREQU	27118.64 Hz
FLT	13550 Hz
DELAY	14.80 usec
ACQTM	1.2083 sec
PD	1.7920 sec
ADBIT	16
RGAIN	25
BF	0.10 Hz
T1	0.00
T2	0.00
T3	90.00
T4	100.00
EXMOD	BCM
EXPCM	Bilevel.complete.decoupling:Set_IRRF
IRNUC	1H
IFR	399.65 MHz
IRSET	124.00 KHz
IRFIN	10500.00 Hz
IRRPW	45 usec
IRATN	511
DFILE	Rxn481 c-Hex カラム C .als
SF	TH5ATFG2
LKSET	61.60 KHz
LKFIN	79.0 Hz
LKLEV	180
LGAIN	24
LKPHS	240
LKSIG	1197
CSPED	13 Hz
FILDC	
FILDF	

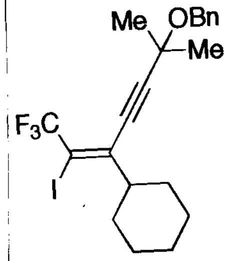

auto

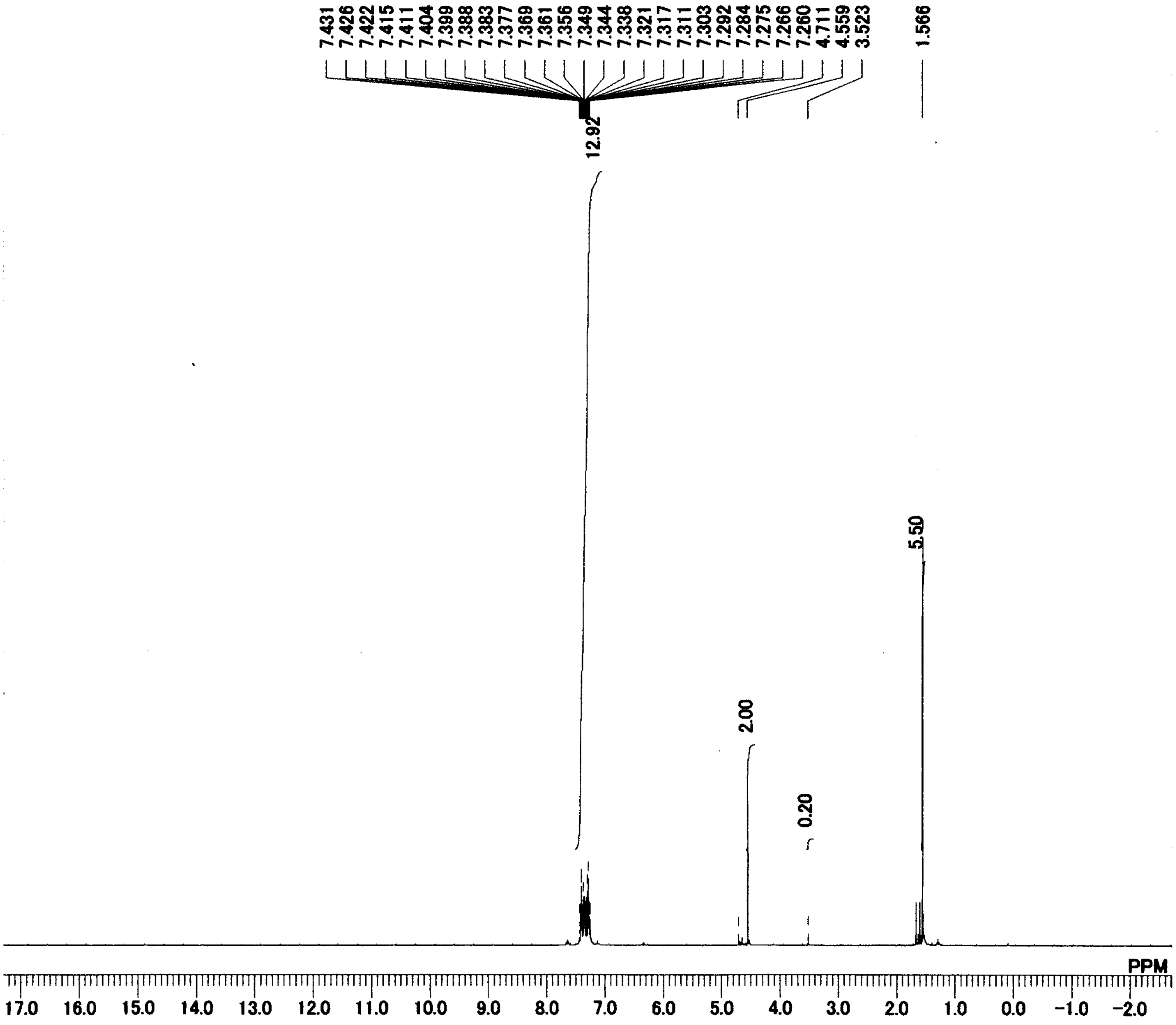

MENUF	1H
OBNUC	1H
OFR	399.65 MHz
OBSET	135.40 KHz
OBFIN	24.90 Hz
PW1	5.50 usec
DEADT	72.20 usec
PREDL	0.20000 msec
IWT	1.0000 msec
POINT	16384
SPO	16384
TIMES	8
DUMMY	1
FREQU	7992.01 Hz
FLT	4000 Hz
DELAY	50.00 usec
ACQTM	2.0501 sec
PD	4.9500 sec
ADBIT	16
RGAIN	13
BF	0.10 Hz
T1	0.00
T2	0.00
T3	90.00
T4	100.00
EXMOD	NON
EXPCM	NON:Single.coupled:PW1_ACQTM_PD
IRNUC	1H
IFR	399.65 MHz
IRSET	136.90 KHz
IRFIN	97.50 Hz
IRRPW	45 usec
IRATN	511
DFILE	Rxn480 カラム H.als
SF	TH5ATFG2
LKSET	61.60 KHz
LKFIN	79.0 Hz
LKLEV	180
LGAIN	24
LKPHS	240
LKSIG	1274
CSPED	14 Hz
FILDC	
FILDF	

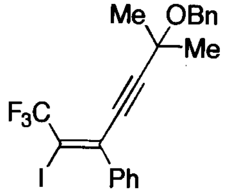

auto

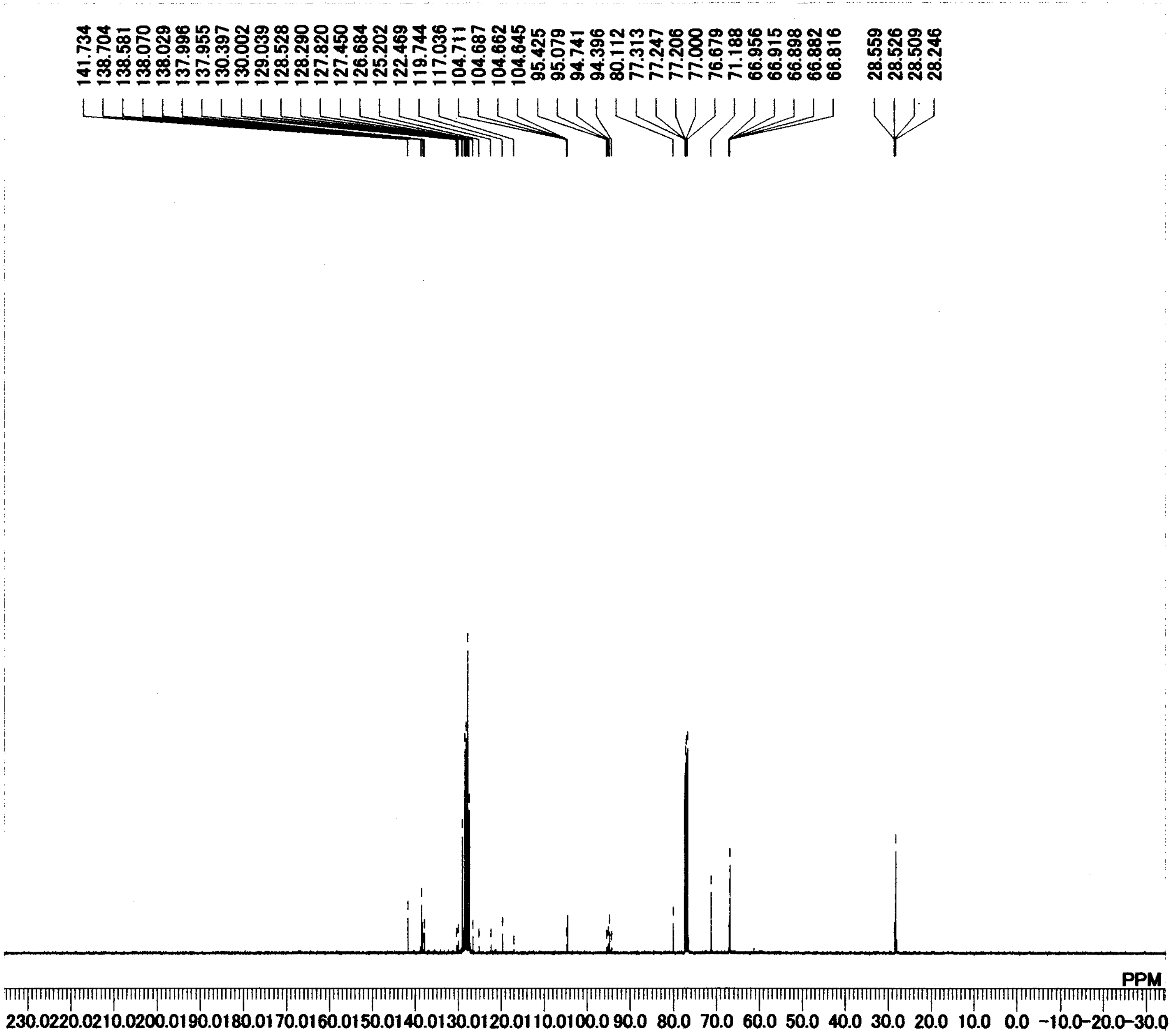

MENUF	13C
OBNUC	13C
OFR	100.40 MHz
OBSET	125.00 KHz
OBFIN	10500.00 Hz
PW1	6.20 usec
DEADT	19.00 usec
PREDL	0.20000 msec
IWT	1.0000 msec
POINT	32768
SPO	32768
TIMES	20000
DUMMY	1
FREQU	27118.64 Hz
FLT	13550 Hz
DELAY	14.80 usec
ACQTM	1.2083 sec
PD	1.7920 sec
ADBIT	16
RGAIN	25
BF	0.10 Hz
T1	0.00
T2	0.00
T3	90.00
T4	100.00
EXMOD	BCM
EXPCM	Bilevel.complete.decoupling:Set_IRRF
IRNUC	1H
IFR	399.65 MHz
IRSET	124.00 KHz
IRFIN	10500.00 Hz
IRRPW	45 usec
IRATN	511
DFILE	Rxn.480 13C.als
SF	TH5ATFG2
LKSET	61.60 KHz
LKFIN	79.0 Hz
LKLEV	180
LGAIN	24
LKPHS	240
LKSIG	1243
CSPED	14 Hz
FILDC	
FILDF	

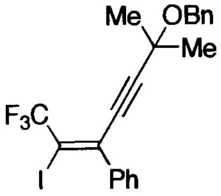

auto

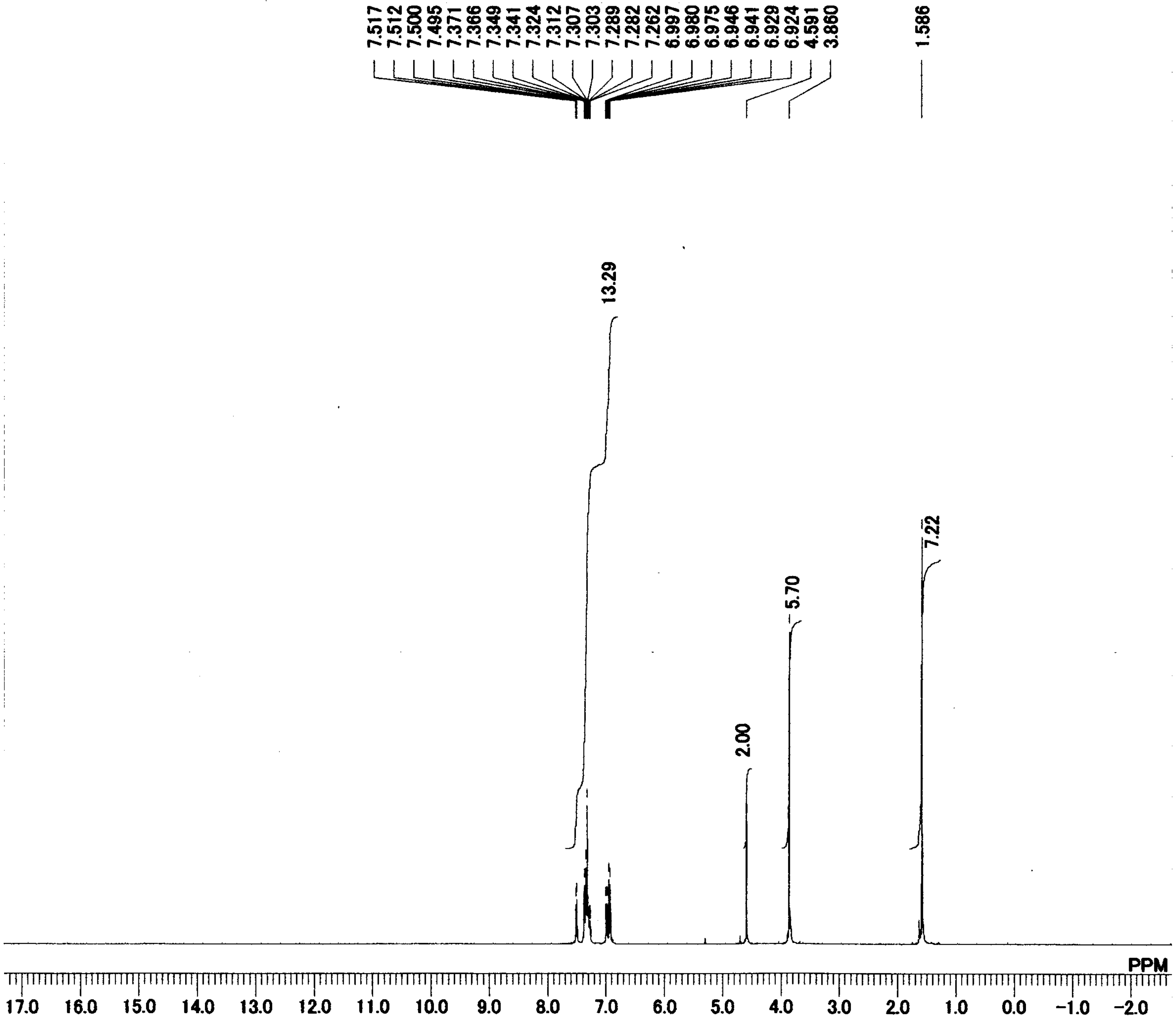

MENUF	1H
OBNUC	1H
OFR	399.65 MHz
OBSET	135.40 KHz
OBFIN	24.90 Hz
PW1	5.50 usec
DEADT	72.20 usec
PREDL	0.20000 msec
IWT	1.0000 msec
POINT	16384
SPO	16384
TIMES	8
DUMMY	1
FREQU	7992.01 Hz
FLT	4000 Hz
DELAY	50.00 usec
ACQTM	2.0501 sec
PD	4.9500 sec
ADBIT	16
RGAIN	12
BF	0.10 Hz
T1	0.00
T2	0.00
T3	90.00
T4	100.00
EXMOD	NON
EXPCM	NON:Single.coupled:PW1_ACQTM_PI
IRNUC	1H
IFR	399.65 MHz
IRSET	136.90 KHz
IRFIN	97.50 Hz
IRRPW	45 usec
IRATN	511
DFILE	Rxn483 PMP カラム H.als
SF	TH5ATFG2
LKSET	61.60 KHz
LKFIN	79.0 Hz
LKLEV	180
LGAIN	25
LKPHS	240
LKSIG	1200
CSPED	12 Hz
FILDC	
FILDF	

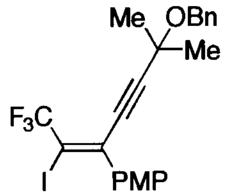

auto

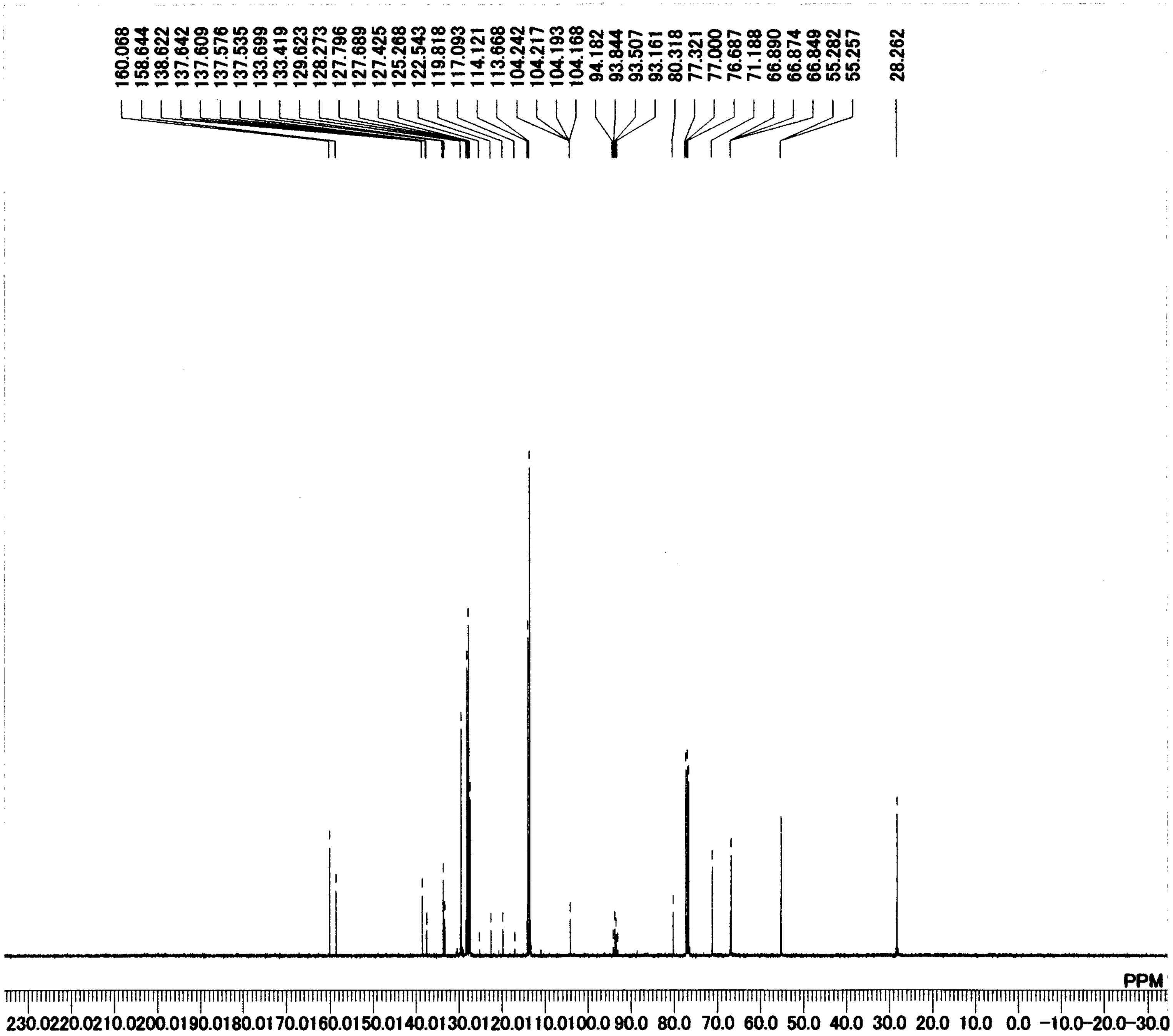

MENUF	13C
OBNUC	13C
OFR	100.40 MHz
OBSET	125.00 KHz
OBFIN	10500.00 Hz
PW1	6.20 usec
DEADT	19.00 usec
PREDL	0.20000 msec
IWT	1.0000 msec
POINT	32768
SPO	32768
TIMES	10000
DUMMY	1
FREQU	27118.64 Hz
FLT	13550 Hz
DELAY	14.80 usec
ACQTM	1.2083 sec
PD	1.7920 sec
ADBIT	16
RGAIN	25
BF	0.10 Hz
T1	0.00
T2	0.00
T3	90.00
T4	100.00
EXMOD	BCM
EXPCM	Bilevel.complete.decoupling:Set_IRRF
IRNUC	1H
IFR	399.65 MHz
IRSET	124.00 KHz
IRFIN	10500.00 Hz
IRRPW	45 usec
IRATN	511
DFILE	Rxn483 PMP カラム C.als
SF	TH5ATFG2
LKSET	61.60 KHz
LKFIN	79.0 Hz
LKLEV	180
LGAIN	24
LKPHS	240
LKSIG	997
CSPED	12 Hz
FILDC	
FILDF	

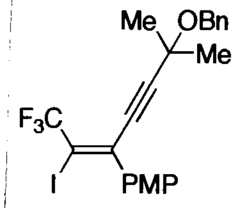

auto

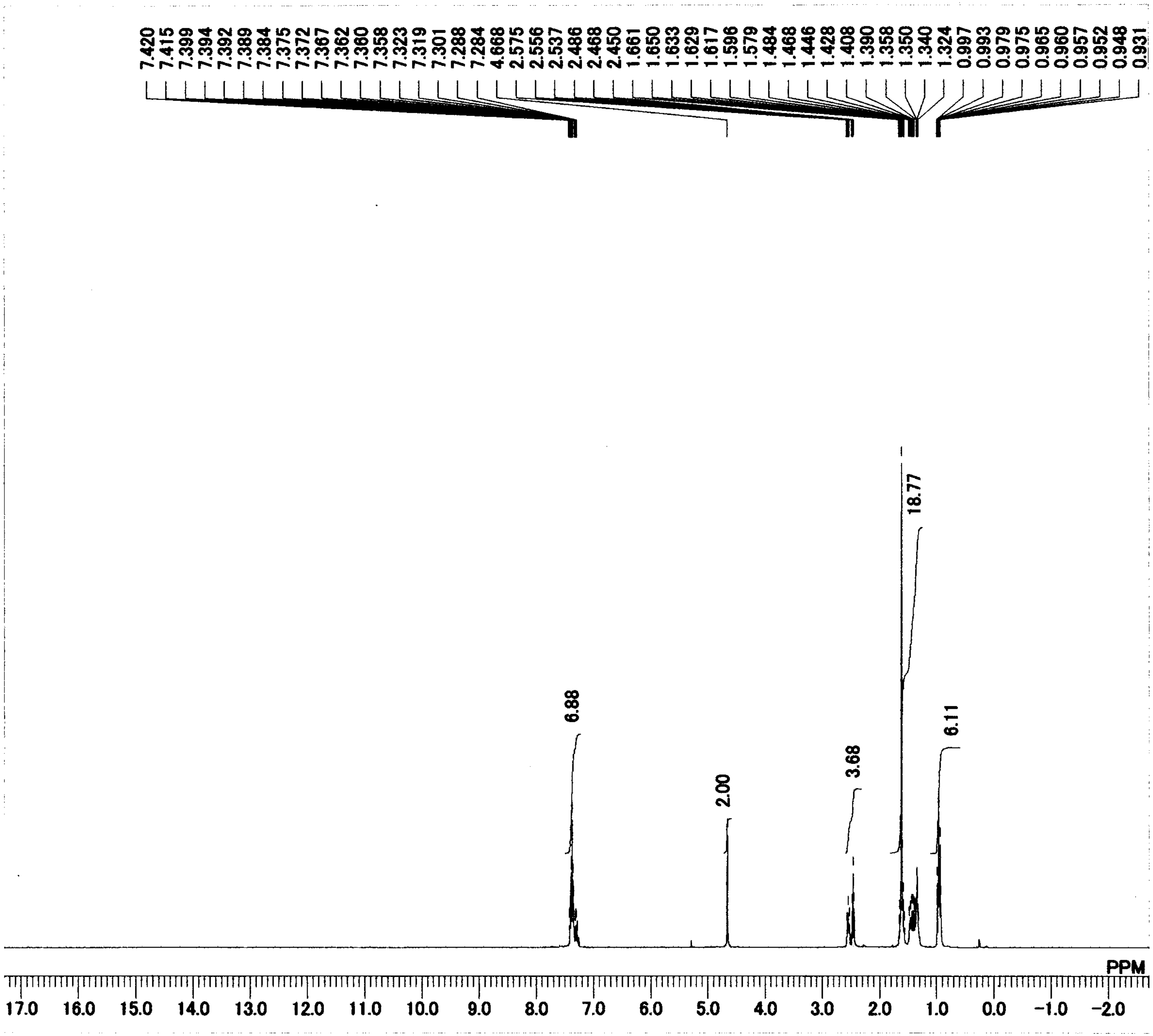

MENUF	1H
OBNUC	1H
OFR	399.65 MHz
OBSET	135.40 KHz
OBFIN	24.90 Hz
PW1	5.50 usec
DEADT	72.20 usec
PREDL	0.20000 msec
IWT	1.0000 msec
POINT	16384
SPO	16384
TIMES	8
DUMMY	1
FREQU	7992.01 Hz
FLT	4000 Hz
DELAY	50.00 usec
ACQTM	2.0501 sec
PD	4.9500 sec
ADBIT	16
RGAIN	8
BF	0.00 Hz
T1	0.00
T2	0.00
T3	90.00
T4	100.00
EXMOD	NON
EXPCM	NON:Single.coupled:PW1_ACQTM_PD
IRNUC	1H
IFR	399.65 MHz
IRSET	136.90 KHz
IRFIN	97.50 Hz
IRRPW	45 usec
IRATN	511
DFILE	Rxn463 sonogashira H.als
SF	TH5ATFG2
LKSET	61.60 KHz
LKFIN	79.0 Hz
LKLEV	180
LGAIN	26
LKPHS	240
LKSIG	1692
CSPED	13 Hz
FILDC	
FILDF	

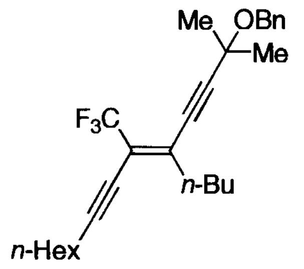

auto

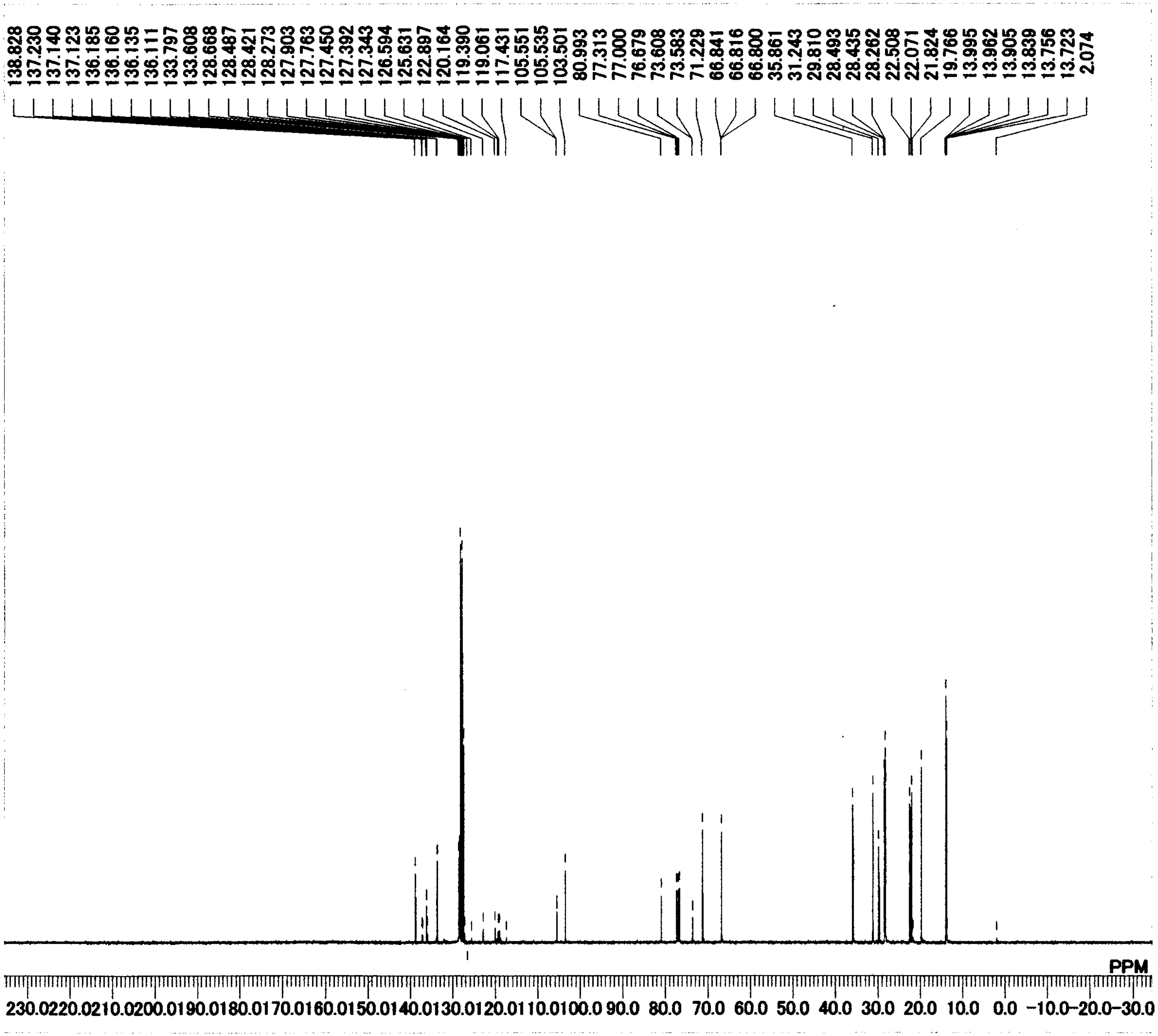

MENUF	13C
OBNUC	13C
OFR	100.40 MHz
OBSET	125.00 KHz
OBFIN	10500.00 Hz
PW1	6.20 usec
DEADT	19.00 usec
PREDL	0.20000 msec
IWT	1.0000 msec
POINT	32768
SPO	32768
TIMES	2000
DUMMY	1
FREQU	27118.64 Hz
FLT	13550 Hz
DELAY	14.80 usec
ACQTM	1.2083 sec
PD	1.7920 sec
ADBIT	16
RGAIN	24
BF	0.00 Hz
T1	0.00
T2	0.00
T3	90.00
T4	100.00
EXMOD	BCM
EXPCM	Bilevel.complete.decoupling:Set_IRRF
IRNUC	1H
IFR	399.65 MHz
IRSET	124.00 KHz
IRFIN	10500.00 Hz
IRRPW	45 usec
IRATN	511
DFILE	Rxn463 sonogashira C.als
SF	TH5ATFG2
LKSET	61.60 KHz
LKFIN	79.0 Hz
LKLEV	180
LGAIN	24
LKPHS	240
LKSIG	967
CSPED	13 Hz
FILDC	
FILDF	

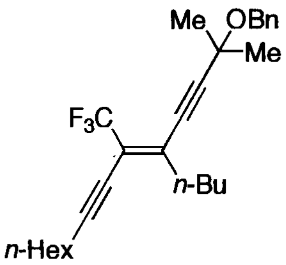

auto

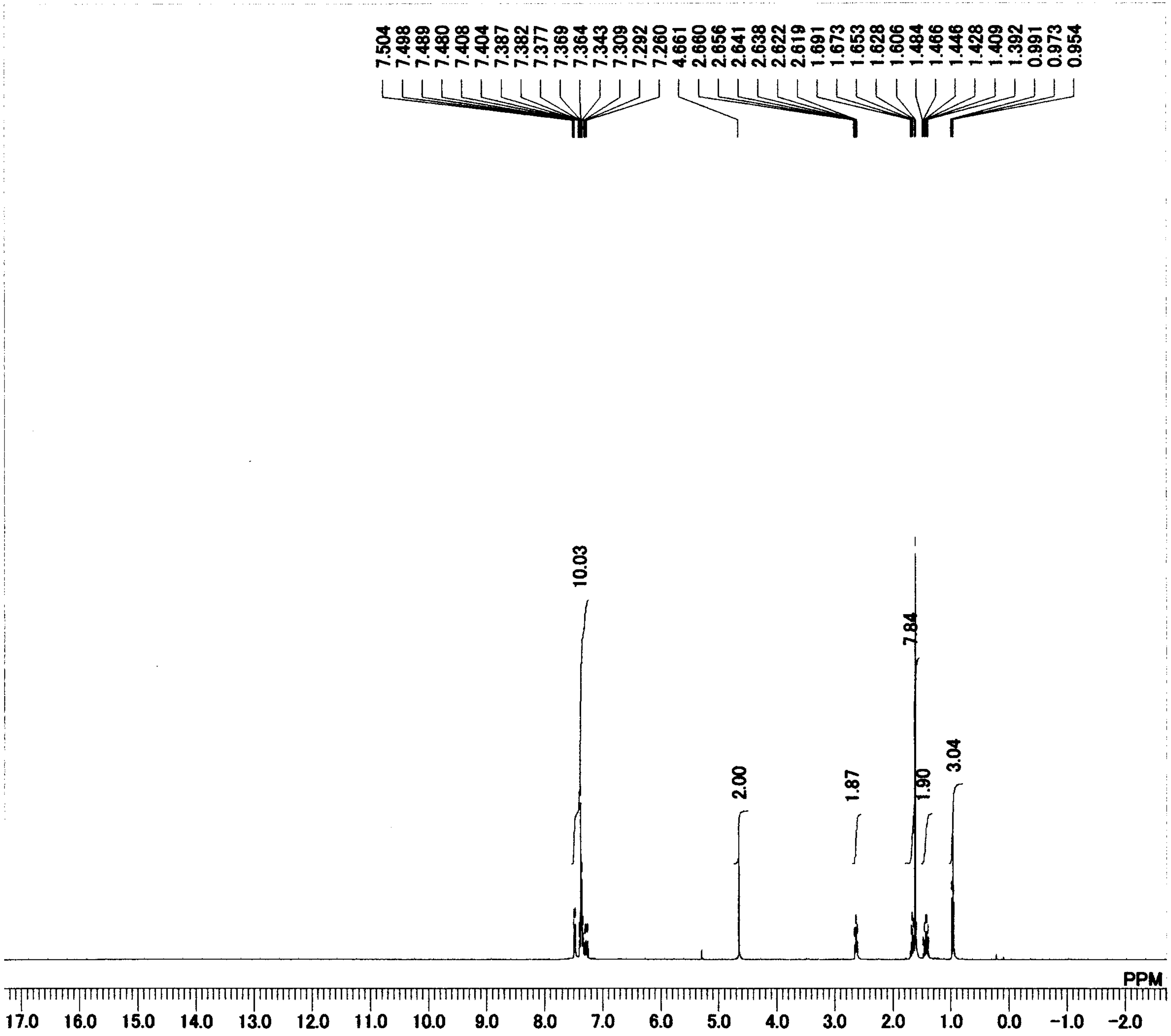

MENUF	1H
OBNUC	1H
OFR	399.65 MHz
OBSET	135.40 KHz
OBFIN	24.90 Hz
PW1	5.50 usec
DEADT	72.20 usec
PREDL	0.20000 msec
IWT	1.0000 msec
POINT	16384
SPO	16384
TIMES	8
DUMMY	1
FREQU	7992.01 Hz
FLT	4000 Hz
DELAY	50.00 usec
ACQTM	2.0501 sec
PD	4.9500 sec
ADBIT	16
RGAIN	13
BF	0.10 Hz
T1	0.00
T2	0.00
T3	90.00
T4	100.00
EXMOD	NON
EXPCM	NON:Single.coupled:PW1_ACQTM_P[illegible]
IRNUC	1H
IFR	399.65 MHz
IRSET	136.90 KHz
IRFIN	97.50 Hz
IRRPW	45 usec
IRATN	511
DFILE	Rxn471 sonogashira H.als
SF	TH5ATFG2
LKSET	61.60 KHz
LKFIN	79.0 Hz
LKLEV	180
LGAIN	23
LKPHS	240
LKSIG	884
CSPED	14 Hz
FILDC	
FILDF	

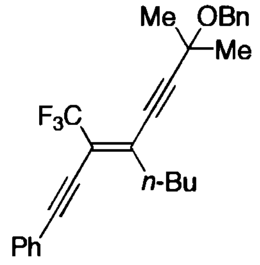

auto

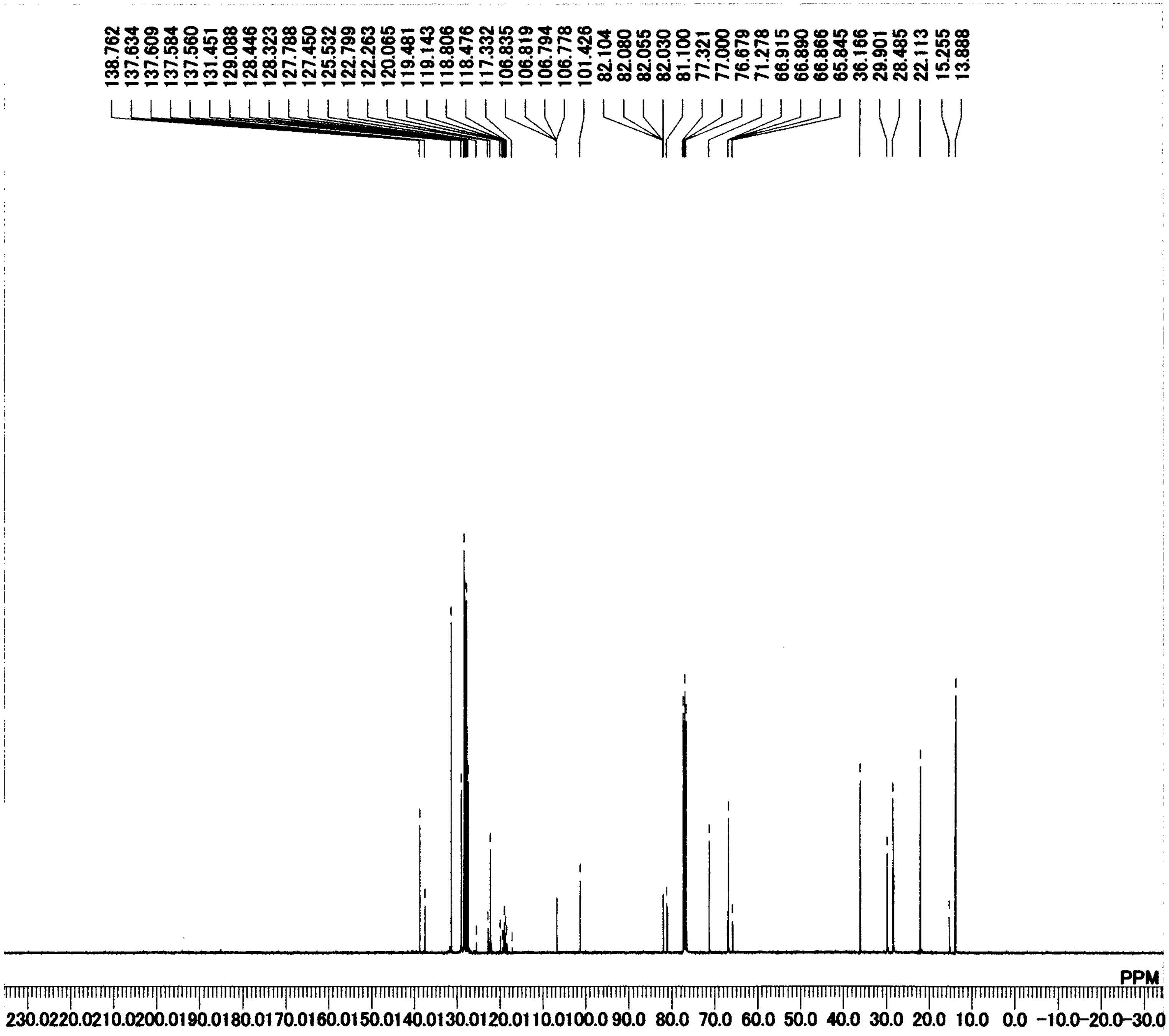

MENUF	13C
OBNUC	13C
OFR	100.40 MHz
OBSET	125.00 KHz
OBFIN	10500.00 Hz
PW1	6.20 usec
DEADT	19.00 usec
PREDL	0.20000 msec
IWT	1.0000 msec
POINT	32768
SPO	32768
TIMES	20000
DUMMY	1
FREQU	27118.64 Hz
FLT	13550 Hz
DELAY	14.80 usec
ACQTM	1.2083 sec
PD	1.7920 sec
ADBIT	16
RGAIN	25
BF	0.10 Hz
T1	0.00
T2	0.00
T3	90.00
T4	100.00
EXMOD	BCM
EXPCM	Bilevel.complete.decoupling:Set_IRRF
IRNUC	1H
IFR	399.65 MHz
IRSET	124.00 KHz
IRFIN	10500.00 Hz
IRRPW	45 usec
IRATN	511
DFILE	Rxn471 sonogashira C.als
SF	TH5ATFG2
LKSET	61.60 KHz
LKFIN	79.0 Hz
LKLEV	180
LGAIN	23
LKPHS	240
LKSIG	866
CSPED	11 Hz
FILDC	
FILDF	

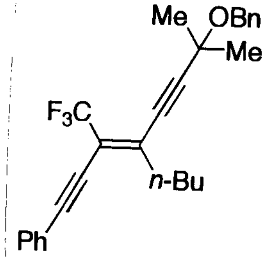

auto

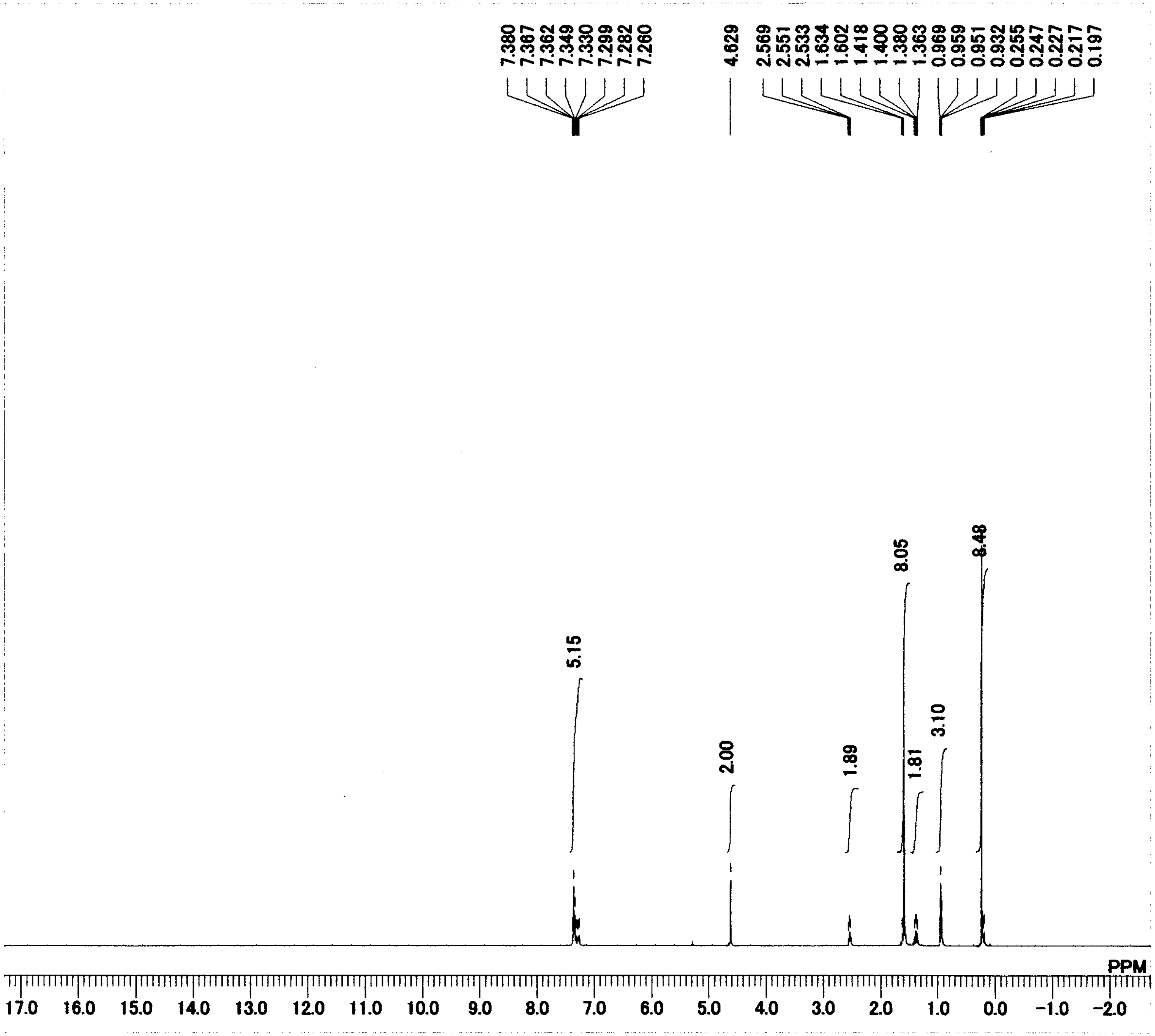

MENUF	1H
OBNUC	1H
OFR	399.65 MHz
OBSET	135.40 KHz
OBFIN	24.90 Hz
PW1	5.50 usec
DEADT	72.20 usec
PREDL	0.20000 msec
IWT	1.0000 msec
POINT	16384
SPO	16384
TIMES	8
DUMMY	1
FREQU	7992.01 Hz
FLT	4000 Hz
DELAY	50.00 usec
ACQTM	2.0501 sec
PD	4.9500 sec
ADBIT	16
RGAIN	11
BF	0.10 Hz
T1	0.00
T2	0.00
T3	90.00
T4	100.00
EXMOD	NON
EXPCM	NON:Single.coupled:PW1_ACQTM_PE
IRNUC	1H
IFR	399.65 MHz
IRSET	136.90 KHz
IRFIN	97.50 Hz
IRRPW	45 usec
IRATN	511
DFILE	Rxn472 カラム H.als
SF	TH5ATFG2
LKSET	61.60 KHz
LKFIN	79.0 Hz
LKLEV	180
LGAIN	24
LKPHS	240
LKSIG	1186
CSPED	12 Hz
FILDC	
FILDF	

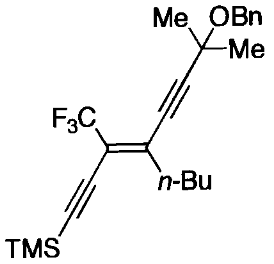

auto

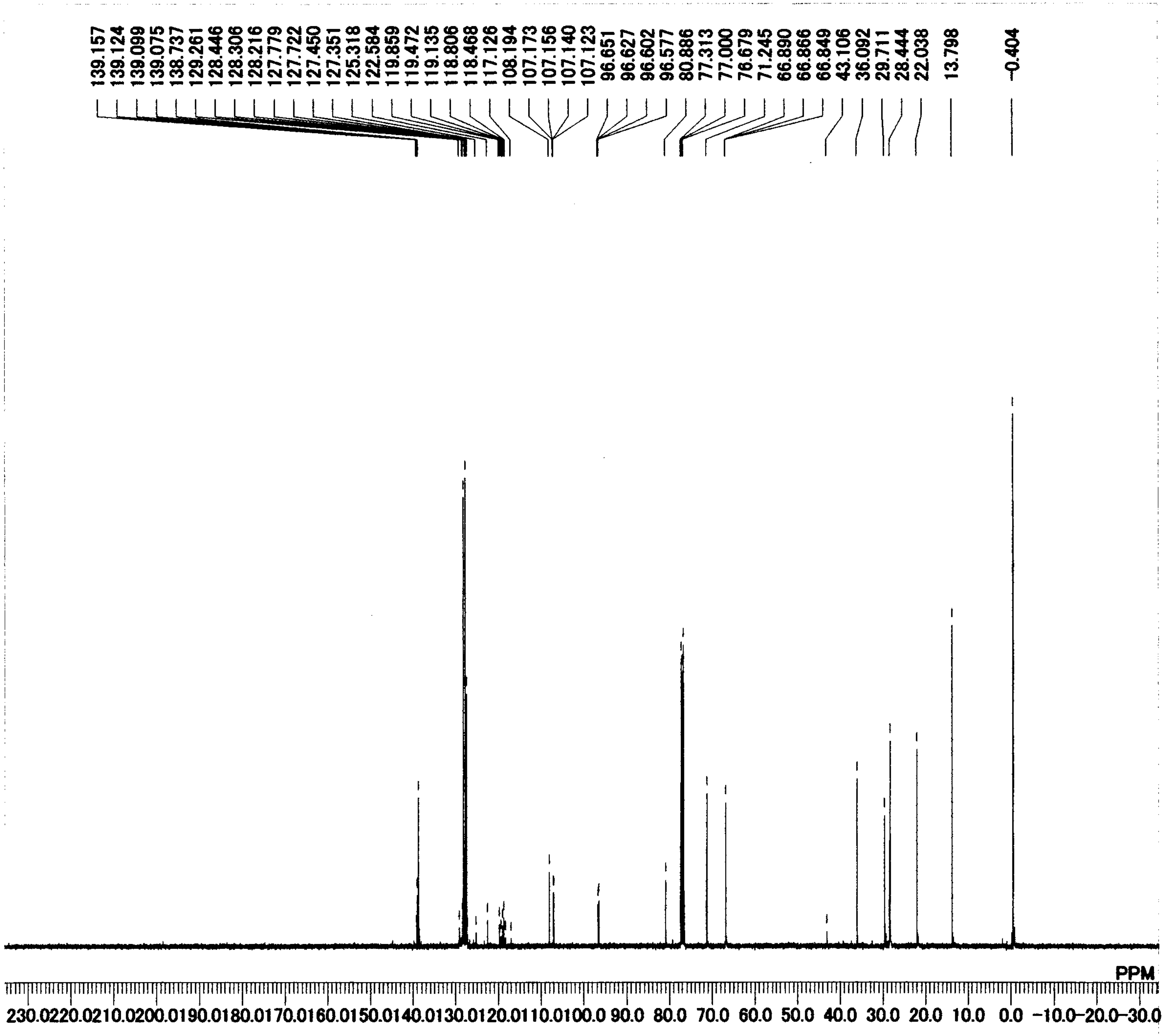

MENUF	13C
OBNUC	13C
OFR	100.40 MHz
OBSET	125.00 KHz
OBFIN	10500.00 Hz
PW1	6.20 usec
DEADT	19.00 usec
PREDL	0.20000 msec
IWT	1.0000 msec
POINT	32768
SPO	32768
TIMES	3000
DUMMY	1
FREQU	27118.64 Hz
FLT	13550 Hz
DELAY	14.80 usec
ACQTM	1.2083 sec
PD	1.7920 sec
ADBIT	16
RGAIN	25
BF	0.10 Hz
T1	0.00
T2	0.00
T3	90.00
T4	100.00
EXMOD	BCM
EXPCM	Bilevel.complete.decoupling:Set_IRRF
IRNUC	1H
IFR	399.65 MHz
IRSET	124.00 KHz
IRFIN	10500.00 Hz
IRRPW	45 usec
IRATN	511
DFILE	Rxn472 カラム C.als
SF	TH5ATFG2
LKSET	61.60 KHz
LKFIN	79.0 Hz
LKLEV	180
LGAIN	24
LKPHS	240
LKSIG	1147
CSPED	13 Hz
FILDC	
FILDF	

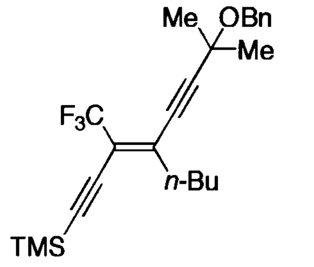

Supplement: File 1 — Experimental, characterization details, and NMR spectra of synthesized compounds, 4e, 13a–e, and 14a–c. [file Beilstein_J_Org_Chem-08-2207-s001.pdf]
